# Ginkgo biloba induces different gene expression signatures and oncogenic pathways in malignant and non-malignant cells of the liver

**DOI:** 10.1371/journal.pone.0209067

**Published:** 2018-12-21

**Authors:** Carolin Czauderna, Mayrel Palestino-Dominguez, Darko Castven, Diana Becker, Luis Zanon-Rodriguez, Jovana Hajduk, Friederike L. Mahn, Monika Herr, Dennis Strand, Susanne Strand, Stefanie Heilmann-Heimbach, Luis E. Gomez-Quiroz, Marcus A. Wörns, Peter R. Galle, Jens U. Marquardt

**Affiliations:** 1 Department of Medicine I, Johannes Gutenberg University, Mainz, Germany; 2 Lichtenberg Research Group “Molecular Hepatocarcinogenesis”, Mainz, Germany; 3 Departamento de Ciencias de la Salud, División de Ciencias Biológicas y de la Salud, Universidad Autónoma Metropolitana-Iztapalapa, Iztapalapa, Mexico; 4 Department of Genomics of Institute of Human Genetics, Life & Brain Center, University of Bonn, Bonn, Germany; Duke University School of Medicine, UNITED STATES

## Abstract

Ginkgo biloba (EGb761) is a widely used botanical drug. Several reports indicate that EGb761 confers preventive as well as anti-tumorigenic properties in a variety of tumors, including hepatocellular carcinoma (HCC). We here evaluate functional effects and molecular alterations induced by EGb761 in hepatoma cells and non-malignant hepatocytes. Hepatoma cell lines, primary human HCC cells and immortalized human hepatocytes (IH) were exposed to various concentrations (0–1000 μg/ml) of EGb761. Apoptosis and proliferation were evaluated after 72h of EGb761 exposure. Response to oxidative stress, tumorigenic properties and molecular changes were further investigated. While anti-oxidant effects were detected in all cell lines, EGb761 promoted anti-proliferative and pro-apoptotic effects mainly in hepatoma cells. Consistently, EGb761 treatment caused a significant reduction in colony and sphere forming ability in hepatoma cells and no mentionable changes in IH. Transcriptomic changes involved oxidative stress response as well as key oncogenic pathways resembling Nrf2- and mTOR signaling pathway. Taken together, EGb761 induces differential effects in non-transformed and cancer cells. While treatment confers protective effects in non-malignant cells, EGb761 significantly impairs tumorigenic properties in cancer cells by affecting key oncogenic pathways. Results provide the rational for clinical testing of EGb761 in preventive and therapeutic strategies in human liver diseases.

## Introduction

Hepatocellular carcinoma is the third leading cause of cancer-related death in men and the fifth in women and shows an increasing incidence in the Western world.[1] The majority of HCCs develop in the background of a chronic inflammatory liver damage subsequently leading to liver cirrhosis.[2] In this context, several predisposing risk factors, such as chronic viral hepatitis, alcohol abuse and metabolic disorders have been identified to promote HCC development, e.g. by increased production of oxidative stress.[3] The constant tissue remodeling and inflammation further enhance intra- and inter-tumor heterogeneity characteristic for HCCs.[4] In line with this, it has also been shown, that the combination of driving oncogenes and type of underlying changes in the hepatic microenvironment define the tumor phenotype highlighting the importance of preventive approaches in clinical management of liver diseases.[5] Recently, it has been reported that anti-oxidant properties of Ginkgo biloba induce hepatoprotective effects in non-malignant liver injuries [6–9] as well as preventive effects against liver tumor initiation.[10] Ginkgo biloba extract is an herbal supplement obtained from the leaves of the ginkgo tree. Ginkgo has been extensively administrated over centuries in traditional Chinese medicine.[11] Due to its anti-oxidant and cytoprotective properties it is currently one of the most widely used botanical compounds worldwide. It is administrated for the prevention and treatment of a variety of diseases such as cognitive function disorders, peripheral blood flow insufficiency, tinnitus and vertigo.[12–15] EGb761 is a well-defined standard Ginkgo biloba extract containing 22–24% flavone glycosides (primarily quercetin, kaempferol and isorhamnetin) and 6% terpene lactones (2,8–3,4% ginkgolides A, B and C and 2,6–3,2% bilobalide).[16] The active constituents of EGb761 seem to exert its effects through interaction with multiple molecular mechanisms and signaling pathways. An ERK1/2-signaling and cell cycle control gene-dependent regulation has been proposed in gastric cancer—[17, 18], steroidogenesis pathways and aromatase activity in breast cancer cells [19, 20], the mitochondrial pathway of apoptosis in melanoma—[21] or STAT3-activity in prostate cancer cells [22] has been described. However, the exact molecular mechanisms underlying protective and anti-tumorigenic effects of EGb761 in the liver are not yet fully understood.

Here, we assessed transcriptomic changes of hepatoma cells as well as immortalized hepatocytes (IH) induced by a short-term treatment with EGb761. We confirm that EGb761 possess anti-oxidant as well as anti-tumor properties and show that it acts through a specific deregulation of key oncogenic pathways in cancer cells leading to a differential response in malignant and non-malignant cells of the liver.

## Material and methods

### Cell lines and compounds

Human hepatoma cell lines WRL68, Huh7, immortalized human hepatocyte cell line THLE5B and primary human HCC cells, Pitts1, have been cultured in DMEM, supplemented with 2mM L-glutamine, 1unit/ml penicillin/streptomycin, and 10% FCS at 37°C and 5% CO_2_ as recommended [23–25]. WRL68 cells were obtained from the global bioresource center ATCC, Huh7 from the cell lines service (RIKEN) and Pitts1, a primary human HCC was obtained from a patient undergoing surgery at the UPMC, Pittsburg in accordance with ethical guidelines [26]. THLE5B is a non-neoplastic human hepatocyte cell line generated by transfection of primary human liver epithelial cells with SV40 T antigen [23] and was a gift from Curtis C. Harris. Cells were treated for 24h, 48h and 72h with Ginkgo biloba extract (EGb761) provided by Dr. Wilmar Schwabe GmBH at indicated concentrations.

### Cell proliferation and apoptosis

Cell proliferation was measured by the colorimetric assay (WST-1 based) according to the manufacturer’s protocol (Roche Applied Sciences). 5x10^3^ cells were plated on 96-well plates for 24h followed by administration of EGb761 for 72h with increasing concentrations ranging from 50 to 1000μg/ml. Proliferation was expressed as percent mean change ± SD (n = 4) in treatment compared to control group. Apoptosis was assessed using acridine orange/ethidium bromide approach (Sigma Aldrich: 2μg/ml; green fluorescence, Promega: 2μg/ml; red fluorescenceas) described previously.[25] Quantification of apoptotic cells co-stained with acridine orange and ethidium bromide was performed on the images taken with confocal microscope Zeiss NLO710 as described.[25] Viable (green) and apoptotic (red) cells were counted in five independent images from three replicate experiments taken with confocal microscope (ZEISS LSM 710 NLO).

### Colony formation and matrigel-based sphere assays

Cells were treated for 72h with cell line specific IC50 for each of the hepatoma cell lines and IH were treated with median IC50 (200 μg/ml) of all hepatoma cells to reflect the EGb761 doses required to induce anti-tumor effects. 1x10^3^ cells were plated on 6-well plates for colony formation and 1x10^3^ cells were plated on 48-well plates for sphere assays after resuspending in 100μl of medium and Matrigel (vol/vol) (BD Biosciences, Bedford, MA). Colony and sphere forming potential was calculated at day 10 for colonies and day 14 for spheres and represented as number of colonies/ spheres per seeded cells. All experiments were performed in three independent replicates.

### Detection of changes in redox status

Chlormethyl-2’,7’-dichlordihydrofluoresciein-diacetat (CM-H2DCFDA, Life Technologies, Invitrogen) has been used as a cell-permeable indicator for reactive oxygen species (ROS) as described before.[27] In brief, 1x10^6^ cells were plated in petri-dish and after 24h incubation treated for 72h with cell line specific IC50 concentrations of EGb761 in hepatoma cells and median IC50 concentration in human hepatocytes (THLE5B). For flow cytometer analyses, 500x10^3^ cells were transferred in FACS-tubes and washed once with PBS. After centrifugation for 5 minutes at 1000 RPM, cells were resuspended in 500μl HBSS (Hank’s Balanced Salt Solution, Gibco, ThermoFisher Scientific) without phenol red. For induction of redox status changes, cell lines were treated for 20 minutes at 37°C with lowest concentration of H_2_O_2_ necessary for an increase of mean fluorescence intensity (Huh7: 1μM, WRL68: 50μM, Pitts1: 1000μM, THLE5B: 50μM). Redox status changes were assessed incubating cells with CM-H_2_DCF-DA (Life Technologies, Invitrogen: 10μmol/L) for 45 minutes. After incubation cells were immediately stocked on ice. Cellular viability was assessed by 5μl 7-actinoaminomycin (Life Tecnologies, Invitrogen). A total of 20.000 events were analyzed in flow cytometer (Becton Dickinson LSRFortessa) and fluorescence of DCF was excited with a blue laser (BL488nm) and emission spectrum was detected with a band-pass filter 530/30. All experiments were performed in three independent replicates.

### RNA extraction

Total RNAs was extracted using Qiagen RNEasy mini Kit (Qiagen GmBH, Hilden, Germany) following the manufacturer’s protocol. RNA quantity and purity were estimated using a Nanodrop ND-1000 spectrophotometer (NanoDrop Technologies, Wilmington, DE) and integrity was assessed by Agilent 2100 Bioanalyzer (Agilent, Palo Alto, CA).

### Microarray analysis

A total of 200ng RNA was linearly amplified as recommended by the manufacturer (Ambion, Austin, TX) and analyses were performed as described before.[28] Gene expression values were normalized by quantile normalization method across all samples following subtraction of background noises in each spot by GenomeStudio (illumina). Signal intensity with a detection *P* > 0.05 was treated as a missing value, and only genes with sufficient representation across the samples were included in further data analysis. Differentially expressed genes between treated and untreated cells from the individual cell lines were determined by resampling for the difference of means included in the boot R-package version 1.3–18. *P* <0.05 were considered statistically significant. Hierarchical cluster analyses were based on Pearson correlation, and complete linkage was performed with Cluster 3.0, including a filter of 80% presence for each gene. Results were visualized with TreeView 1.60 (Michael Eisen Laboratory, Lawrence Berkeley National Laboratory and University of California, Berkeley; http://rana.lbl.gov/eisen/). Ingenuity Pathway Analysis (Ingenuity Systems Inc.) tool was used for functional classification and network analyses. The significance of each network, function and pathway was determined by the scoring system provided by Ingenuity Pathway Analysis tool.

### Real-time PCR

A two-step RT-qPCR, cDNA synthesis using SuperscriptIII (Invitrogen), SYBR Green Master-Mix (Bio-Rad) and *iQ5 or CFX Connect* System was performed. Oligonucleotide primers were designed using Primer3 v.0.4.0 (http://frodo.wi.mit.edu/primer3/) as described before [28]. The amplification protocol was as follows: 95°C for 3 min, followed by 40 cycles of 95°C for 15 seconds and 1 minute at 60°C, completed by a dissociation curve to identify false positive amplicons. Glyceraldehyde-3-phosphate dehydrogenase (GAPDH) was used as a reference for THLE5B, Huh7 and WRL68 and hypoxanthine phosphoribosyltransferase 1 (HPRT1) for Pitts1. The relative expression level of each gene was normalized to untreated cells and calculated using the formula 2^(−ΔΔCt)^.

### Western blotting

Monolayer cultures of each cell line were exposed to cell line specific IC50 concentrations of EGb761 in hepatoma cells and to the indicated median IC50 concentration in human hepatocytes for 24h, 48h and 72h. Cell lysates were prepared from frozen cells using M-PER Tissue extraction Buffer (Pierce) containing complete protease inhibitor cocktail (Roche). Protein concentrations have been determined by the BCA protein assay (Thermo Fisher) following the manufacturer’s protocol. 25ug have been used for western blotting; separated by SDS-PAGE and transferred onto nitrocellulose membrane (Hartenstein) as described previously.[29] PageRuler Prestained Protein Ladder (Thermo Fisher Scientific) has been used on the left site and Full-Range Rainbow Molecular Weight Marker (Thermo Fisher Scientific) on the right site of each membrane. Membranes were probed with the indicated antibodies. Antibodies were diluted 1:1000 and included: beta-Actin Clone B43R (mouse, monoclonal, Briovision); KEAP1 (rabbit, monoclonal, cell signaling #8047), Nrf2 (rabbit, monoclonal, cell signaling #12721); ERK (rabbit, monoclonal, cell signaling #4695); phosphor-ERK (rabbit, polyclonal, cell signaling #9101); AKT (rabbit, monoclonal, cell signaling #13038); phosphor-AKT (rabbit, monoclonal, cell signaling #4685); mTOR 7C10 (rabbit, monoclonal, cell signaling #2983); phosphor-mTOR (rabbit, polyclonal, cell signaling #2971). Quantification of expression levels was performed by densitometric analyses using ImageJ on original scanned membranes (S2 Fig). For representative imaging original full-length blots were converted to gray-scaled images. EGb761 treated samples (+) and controls (-) have been cropped into one image for each target and were processed equally by changing brightness and contrast.

### Nrf2 activation

Monolayer cultures of each cell line were treated to cell line specific IC50 concentrations of EGb761 in hepatoma cells and to the indicated median IC50 concentration in human hepatocytes for 24h, 48h and 72h. Nuclear protein concentrations of frozen cell pellets have been extracted using the NE-PER Nuclear and Cytoplasmic Extraction Reagents (Thermo Fisher) following the manufacturer’s protocol. Protein concentrations have been determined by the BCA protein assay (Thermo Fisher) following the manufacturer’s protocol. 20ug of nuclear protein have been used for the Nrf2 Transcription Factor Assay Kit (Colorimetric) (ab207223, Abcam) to detect nuclear Nrf2 activation at the colorimetric readout at OD 450nm following the manufacturer’s protocol. All experiments were performed in three independent replicates.

### Statistics

Statistical analysis was performed using Student’s t-test and one-way ANOVA test for multiple group comparisons. P-values ≤ 0.05 were considered statistically significant. Results are presented as means ± SD. Inhibitory concentration 50 (IC50) for each cell line was determined by non-linear regression analyses based on cell proliferation in WST-1 assay.

## Results

### Ginkgo biloba confers anti-oxidant effects

**Fig 1:**
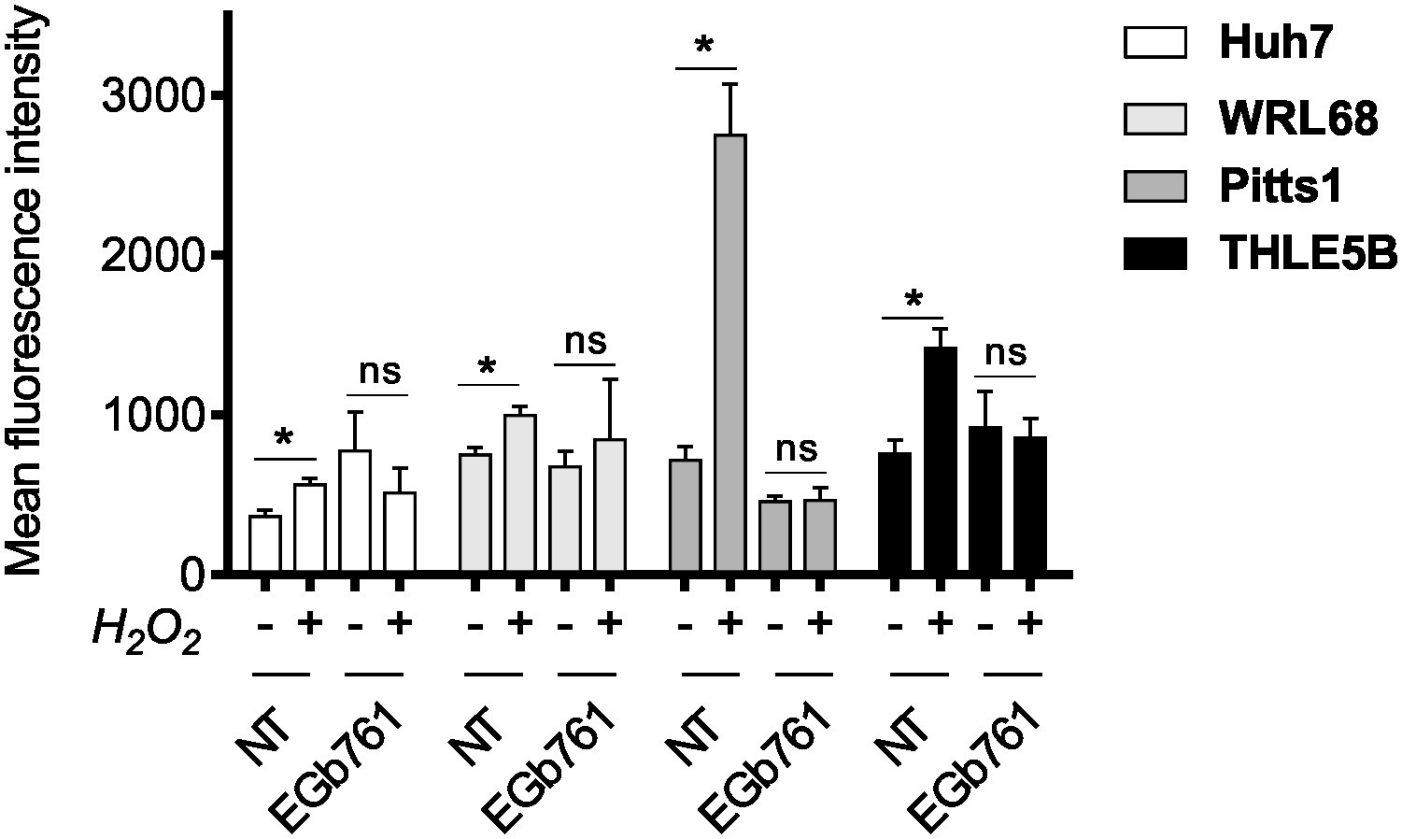
EGb761 exerts anti-oxidant activities. Basal (-) and H_2_O_2_-induced (+) changes in redox status were evaluated by flow cytometry using the ROS-indicator CM-H_2_DCF-DA in non-treated cells (NT) and after 72h exposure to EGb761. The data represent means DCF fluorescence intensity ± SEM of three independent experiments; *p<0.05, analyzed by student’s t-test.

We investigated potential anti-oxidant properties of EGb761 in hepatoma cell lines including one primary HCC cell line referred to as Pitts1 [26] as well as immortalized hepatocytes (IH). Oxidative stress response was evaluated by monitoring the increase of fluorescence of CM-H_2_DCF-DA, an indicator for reactive oxygen species (ROS), by flow-cytometry. We investigated basal as well as induced changes of the redox status by H_2_O_2_ in non-treated and EGb761-treated cells. Cell line specific IC50 concentrations of EGb761 in hepatoma cells and median IC50 concentration in IH were used. The basal status did not differ in EGb761-treated and non-treated (NT) cells. In contrast, after treatment with H_2_O_2_, we observed an increase in mean DCF fluorescence intensity indicating changes in redox status of both non-treated hepatoma cells as well as in non-treated IH. Importantly, EGb761-treatment ameliorated the H_2_O_2_-induced increase in ROS leading to unchanged DCF fluorescence intensities compared to non-treated cells suggesting anti-oxidant properties of the Ginkgo biloba extract (Fig 1).

### Ginkgo biloba confers dose-dependently pro-apoptotic and anti-proliferative effects

**Fig 2:**
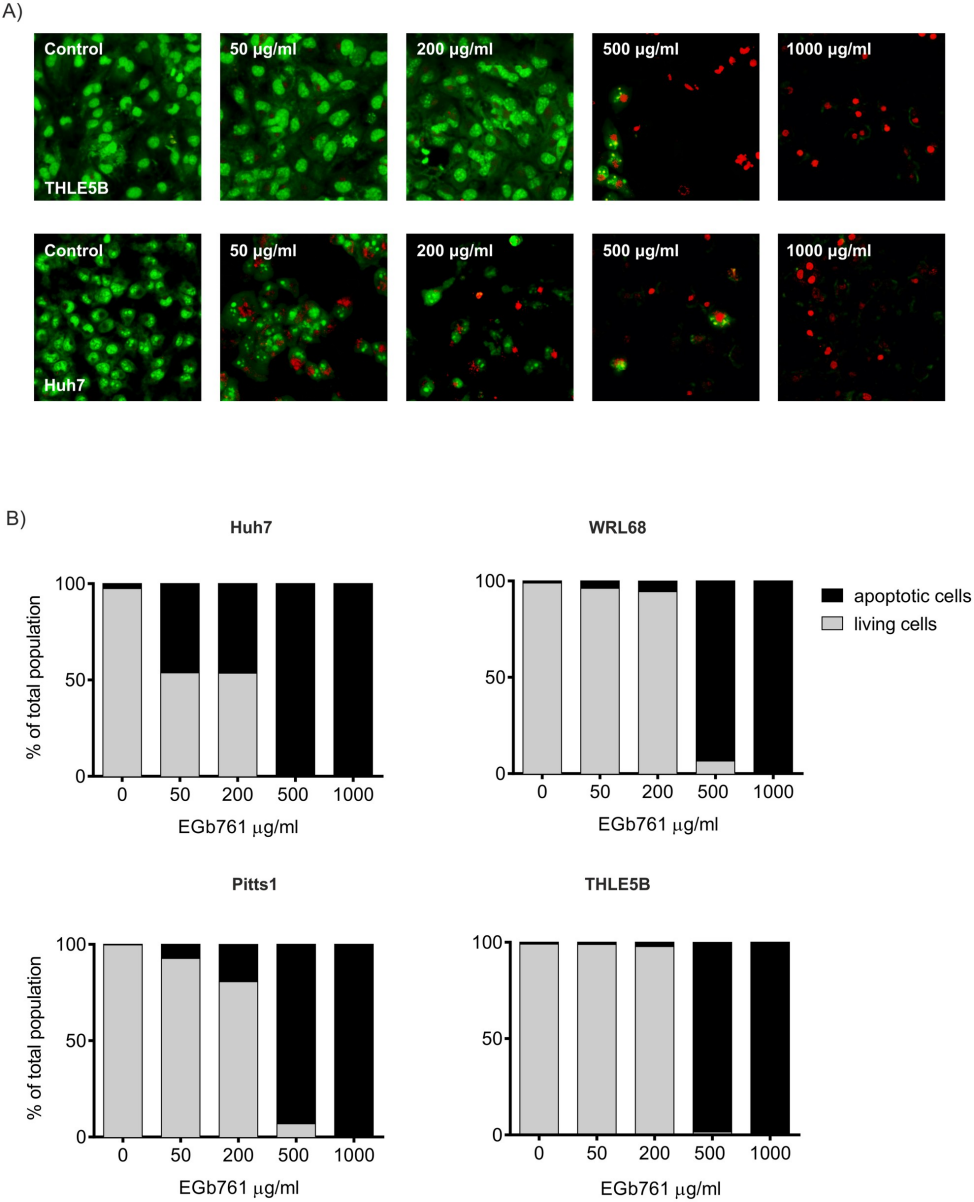
EGb761 induces apoptosis in all cell lines. (A) Representative confocal microscopy of double staining with ethidium bromide (EtBr, red) and acridine orange (AO, green) to quantify apoptosis in the absence or presence of different concentrations (0–1000 μg/ml) of EGb761 for three days in THLE5B and Huh7. (B) Quantification of apoptosis and expressed as a percentage of total population.

**Fig 3:**
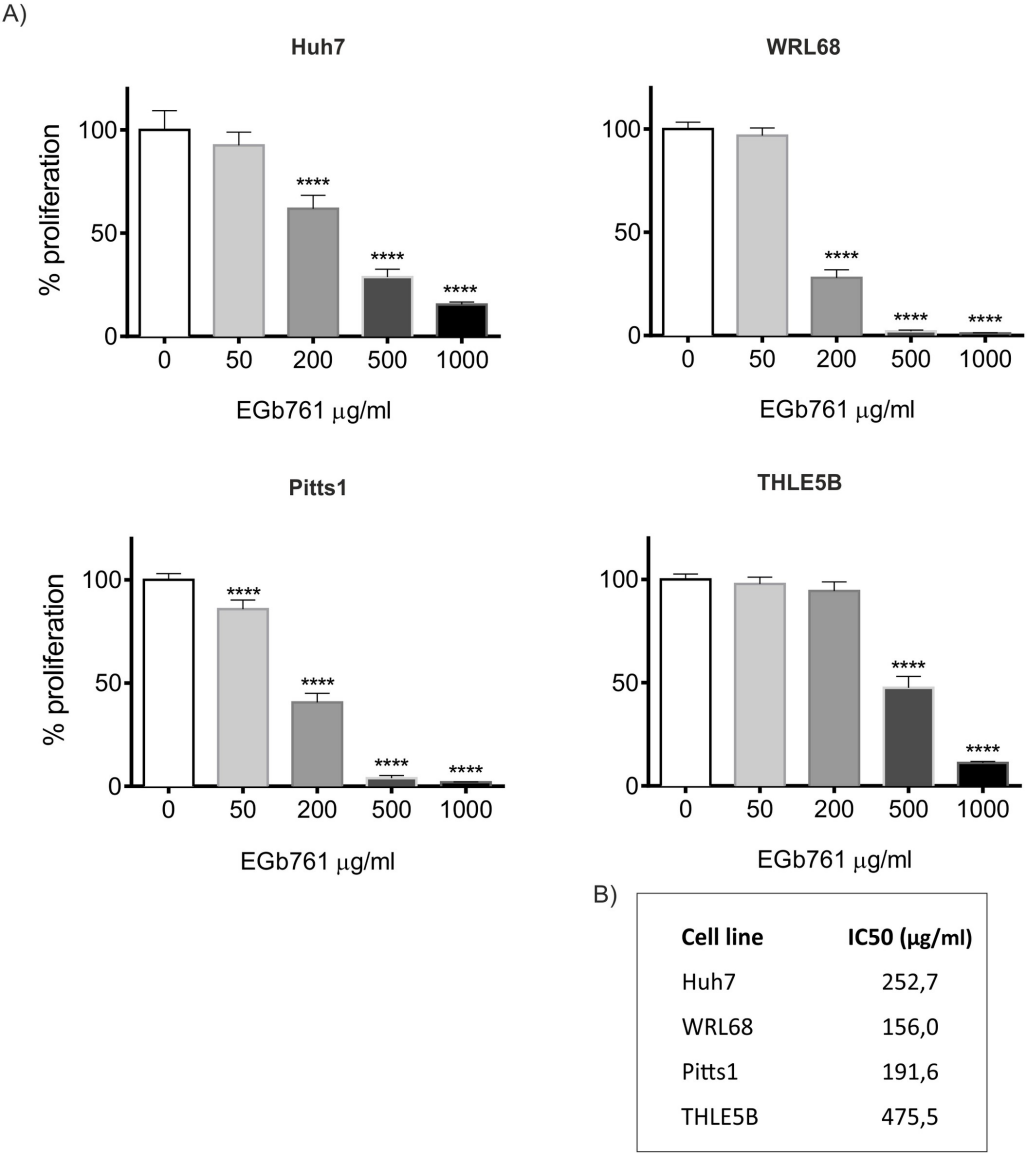
Dose-response of hepatoma cell lines and human hepatocytes to EGb761. (A) Cell proliferation after three-day exposure to indicated concentrations of EGb761 in hepatoma cells and IH using WST-1 assay. The data are means ±SD of four independent experiments, ****p<0.0001, analyzed by one-way ANOVA test for multiple group comparisons. (B) shows the corresponding IC50 values for each cell line.

We next investigated the impact of EGb761 on cell proliferation and apoptosis in three hepatoma cell lines as well as IH. Increasing concentrations of EGb761 ranging from 50 to 1000μg/ml were administered for a total of 72 hours. Double staining with ethidium bromide and acridine orange revealed a considerably higher induction of apoptosis in hepatoma cell lines compared to IH (Fig 2A and 2B). Similarly, EGb761 exposure significantly suppressed cell proliferation of hepatoma cells in a dose-dependent manner. While human hepatoma cells were quite sensitive to the treatment, IH were only affected by high doses of EGb761 (e.g. 500–1000 μg/ml). Accordingly, inhibitory concentration 50 (IC50) of EGb761 was more than twofold higher (IC50_THLE5B_ = 475 μg/ml) for IH compared to IC50 for hepatoma cell lines (IC50_Huh7_ = 252,7μg/ml; IC50_WRL68_ = 156,0μg/ml; IC50_Pitts1_ = 191,6μg/ml) (Fig 3). Together, these results indicate a different sensitivity of malignant and non-malignant cells in the liver to EGb761.

### Gingko biloba impairs tumorigenic potential of hepatoma cell lines

**Fig 4:**
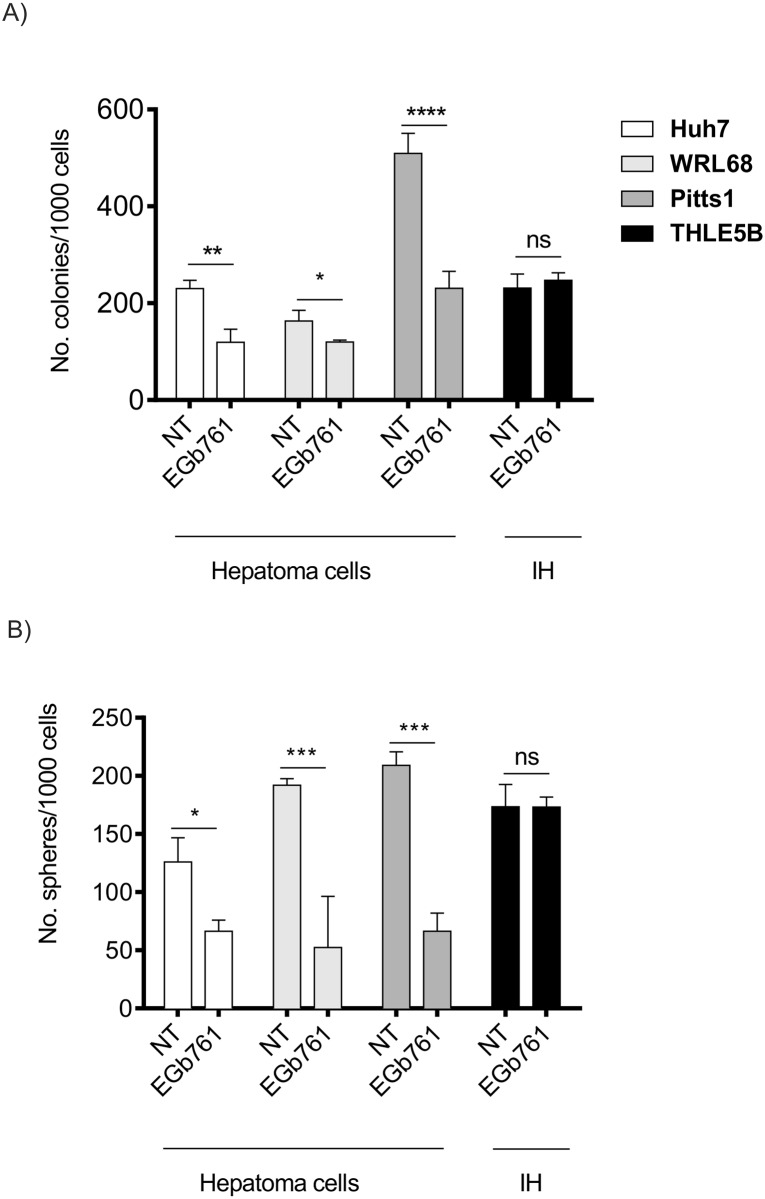
EGb761 reduces colony and sphere forming potential of hepatoma cells. (A) Colony and (B) sphere frequencies after three-day exposure to EGb761. Graphs represent the number of colonies/spheres / 1000 plated cells. The data are means ±SD of three independent experiments; *p<0.05, **p<0.01, ***p<0.001, ****p<0.0001, analyzed by student’s t-test.

Next, we evaluated the impact of EGb761 on functional properties of the cells by investigating the ability of cells to form colonies and spheres after a three-day exposure to EGb761. Cells were treated with respective IC50 for each of the hepatoma cell lines and IH were treated with median IC50 (200 μg/ml) of all hepatoma cells to reflect the EGb761 doses required to induce anti-tumor effects. Consistently, a three-day EGb761 exposure caused a significant reduction in both colony and sphere forming ability in all hepatoma cell lines. Conversely, non-malignant hepatocytes remained unaffected (Fig 4A and 4B). Together these results suggest that the Ginkgo biloba extract, EGb761, exerts anti-oncogenic effects in malignant liver cells, while IH remained unaffected.

### Molecular profiling of Ginkgo biloba effects

**Fig 5:**
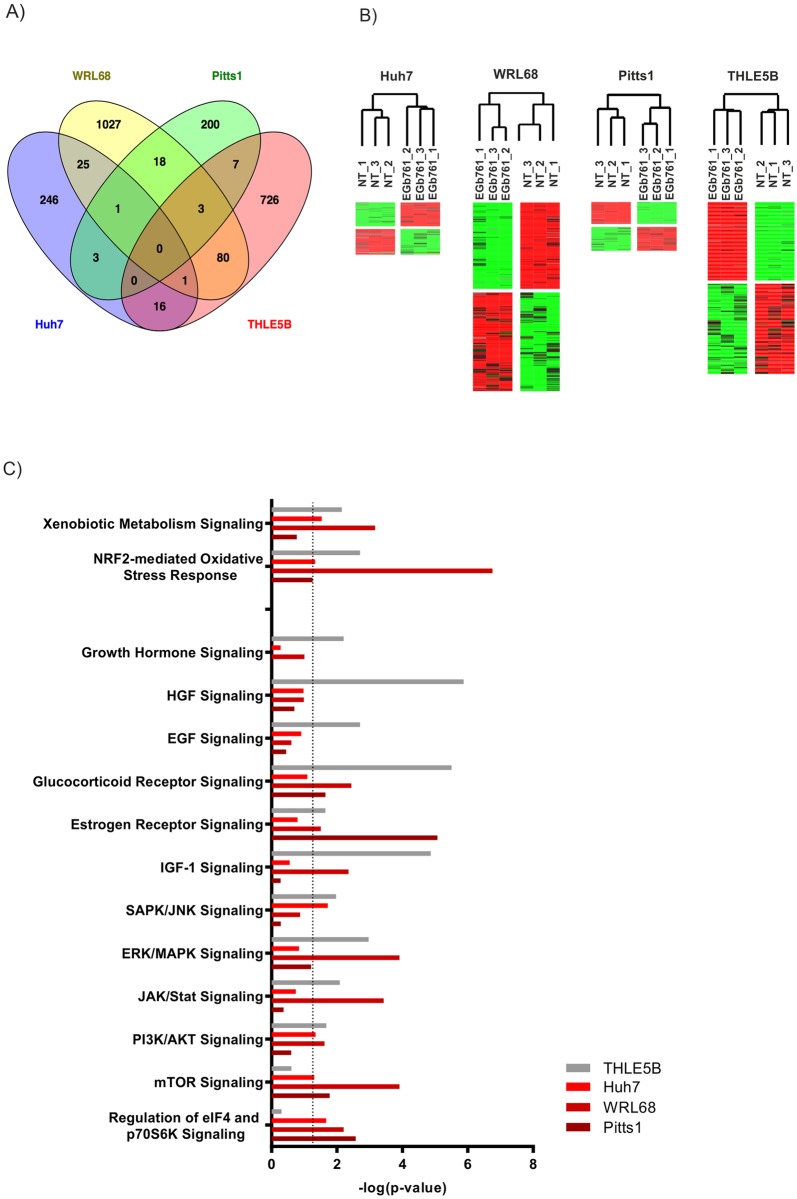
Molecular profiles of EGb761 treated hepatoma cell lines and human hepatocytes. (A) Venn diagram demonstrating the overlap of different gene expression signatures of indicated cell lines of treated (EGb761) versus untreated (NT) cells. (B) Unsupervised hierarchical cluster analysis based on the corresponding significant genes regulated by EGb761. (C) Canonical pathways significantly regulated by EGb761 and identified by Ingenuity Pathway Analysis. Pathways enriched in two or more cell lines (p<0.05) were included and associated to cell proliferation, -growth and -survival and oxidative/metabolic stress response.

To determine potential molecular mechanisms that contribute to the differential effects of EGb761 on normal and malignant cells, we examined global transcriptomic changes for each cell line after a three-day EGb761 treatment by gene expression microarrays. We identified a total of 833 genes in THLE5B (442 up- and 391 down-regulated), 292 genes in Huh7 (149 up- and 143 down-regulated), 1155 genes in WRL68 (544 up- and 611 down-regulated) and 232 genes in Pitts1 (106 up- and 126 down-regulated) differentially expressed after a three-day exposure to EGb761 (p<0,05) (Fig 5A). Unsupervised hierarchical clustering confirmed that identified genes were highly efficient in separating untreated from treated cells in each of the investigated cell lines (Fig 5B). Subsequent network and pathway analyses using Ingenuity Pathway Analysis (IPA) revealed that the major associated networks affected by EGb761 were involved in cell death and survival *(MYC*, *ATG7*, *NOV*, *RANBP1*, *SLK*, *ETS1*, *ZNRF3*, *ZC3H12A*, *ALDH1A3*, *CGRRF1*, *WDR12*), cellular growth and proliferation (*MAP3K8*, *INPPL1*, *RND3*, *ATF1*, *AKR1C3*, *ARIH1*, *NRIP1*, *SESN1*, *VCP*, *TP53BP1*, *HGS*) as well as protein synthesis (*EIF2AK3*, *EIF3L*, *EIF4Aγ*, *EIF4 FKBP1A*, *PSMD2*, *UBE2E1*) (S1 Table). Additionally, xenobiotic metabolism and Nrf2-mediated oxidative stress response as well as key oncogenic signaling pathways resembling ERK/MAPK, IGF-1, PI3K/AKT/mTOR and SAPK/JNK were involved (Fig 5C). Comparative analyses of canonical pathways significantly regulated in at least three of the four cell lines identified six pathways: xenobiotic metabolism signaling and Nrf2-mediated oxidative stress response, PI3K/AKT-signaling and regulation of eIF4 and p70SK6 as well as estrogen—and glucocorticoid receptor signaling. Notably, the regulation of *eIF4* and *p70SK6*, downstream targets of mTOR-signaling pathway, was exclusively affected in malignant cell lines suggesting a potential disruption of cell growth by impaired protein biosynthesis.

### Ginkgo biloba affects Nrf2 and mTOR-signaling pathways

Consistent with our phenotypic investigations, molecular profiles suggest that EGb761 exerts its anti-oxidant and anti-tumor activities by regulating oxidative stress response and key oncogenic pathways. Based on the molecular profiles, we investigated transcriptomic and proteomic changes of the selected pathways after 24h, 48h and 72h of EGb761 treatment.

**Fig 6:**
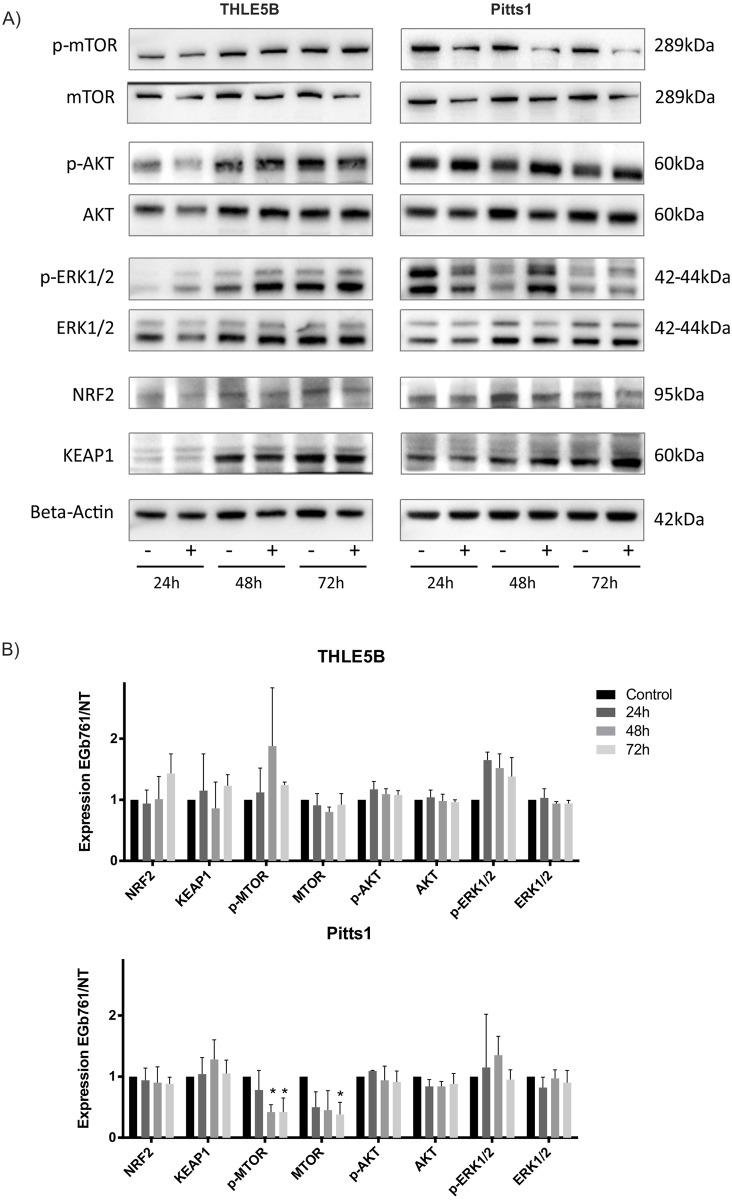
EGb761 modulates oxidative stress response and oncogenic pathways. (A) Western blots for AKT, mTOR, ERK1/2, Nrf2 and KEAP1 in 24h, 48h and 72h treated (+) versus untreated (-) cells are shown in representative images of three independent experiments. (B) Relative expression to control (untreated cells) is demonstrated as means ± SD by quantitative analysis using densitometry normalized to the corresponding beta-actin expression; *p<0.05 analyzed by student’s t-test.

**Fig 7:**
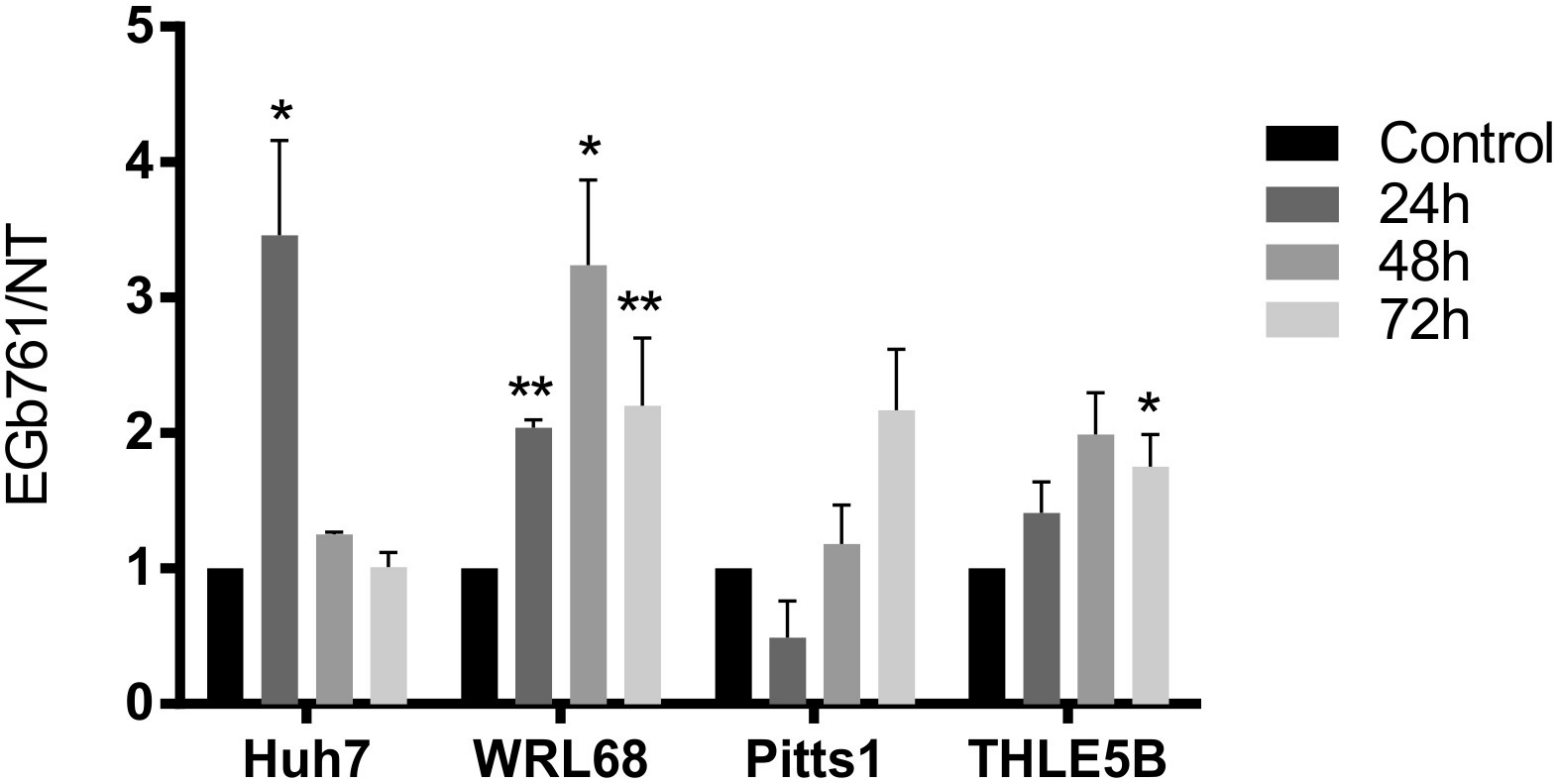
Nrf2 activation by EGb761. Time-dependent relative activation of nuclear Nrf2 in EGb761 treated cells normalized to untreated cells (control) of three independent experiments using the Nrf2 Transcription Factor Assay Kit (Colorimetric, Abcam) is demonstrated as means ± SD. *p<0.05, **p<0.01 analyzed by student’s t-test.

To confirm the impact of EGb761 in oxidative stress response, we assessed expression levels of Nrf2 and KEAP1 by Western Blotting as well as Nrf2 activation by ELISA. While we detected no significant changes for total Nrf2 and KEAP1 (Fig 6 and S1 Fig), we observed a significant nuclear accumulation of Nrf2 by EGb761 in human hepatoma cell lines (Huh7 and WRL68) as well as in IH (THLE5B) (Fig 7), whereby indicating anti-oxidant properties of EGb761 by activation of Nrf2-mediated oxidative stress response.

Furthermore, we investigated key oncogenic pathways induced by EGb761 in both IH and hepatoma cell lines. Western blotting revealed no significant changes in oncogenic pathways in IH, whereas a significant regulation of mTOR signaling could be demonstrated in Pitts1 (Fig 6). Similar trends could be confirmed by time-dependent analyses of selected genes by qRT-PCR analyses in all hepatoma cell lines (S1 Fig). Taken together, these results indicate that EGb761 modulates transcriptional programs related to Nrf2-mediated oxidative stress response as well as oncogenic pathways involved in cell proliferation and growth. The molecular alterations are differentially affected in malignant and non-malignant cells possibly due to deregulation of the signaling pathways during malignant transformation.

## Discussion

Ginkgo biloba is well known for its anti-oxidant as well as anti-atherogenic properties and widely used for a variety of diseases such as neurological disorders, peripheral and central blood flow insufficiencies, tinnitus and vertigo.[11–15] Its role in carcinogenesis is however controversial: While in vivo studies using various Ginkgo biloba extracts reported an increased risk for thyroid cancer and hepatoblastomas in rodents during a long-term treatment as well as an exacerbation of liver metastasis in a mouse colon cancer metastasis model [30, 31], several other studies demonstrated anti-proliferative, apoptosis-inducing and chemopreventive effects of the standardized Ginkgo biloba extract EGb761 in a variety of cancers supporting its use towards cancer prevention and therapy.[17, 18, 21, 22] Herein, we confirm that the standard extract, EGb761, possess anti-oxidant as well as anti-tumorigenic properties on several liver cancer cells and induces multiple molecular changes involved in Nrf2-mediated oxidative and xenobiotic stress response as well as key oncogenic pathways of hepatocarcinogenesis. Importantly, EGb761 differentially affected non-malignant and malignant cells of the liver suggesting that complementary use of EGb761 in patients with chronic liver diseases as well as human HCC patients might be safely possible.

HCC mainly develop on the basis of chronic inflammatory cell death commonly associated with viral hepatitis, alcohol abuse, metabolic syndrome or hereditary liver diseases.[32] The resulting disrupted, inflammatory microenvironment can promote production of reactive oxygen species (ROS) and reactive nitrogen species (RNS), which induce pro-oncogenic mutations and genomic alterations.[33] The Nrf2-mediated oxidative and xenobiotic stress response pathway is one of the most prominent signaling cascade for preventing DNA-damage and mutagenic events. Under homeostatic conditions Nrf2 induces cytoprotective effects whereby preventing excessive cellular dysfunction.[34] Nrf2 is a transcriptional factor and its activity is mainly repressed by KEAP1 in the cytoplasm. Activation by electrophilic compounds or oxidative stress results in a nuclear translocation of Nrf2, where it induces multiple genes of xenobiotic and oxidative stress response.[35] Several reports indicate that Ginkgo biloba at least partly promotes anti-oxidative effects by inducing Keap1-Nrf2-signaling.[36] Consistently, our transcriptomic analyses revealed a strong regulation of downstream targets of xenobiotic and Nrf2-mediated oxidative stress response pathways in both hepatoma cells and hepatocytes (Fig 5C). Nrf2 activation could be confirmed by detecting an accumulation of its nuclear fraction by EGb761 treatment. Functionally, we observed cytoprotective effects of EGb761 in a state of oxidative stress (Fig 1) emphasizing its preventive properties.

In agreement with previous reports, we found that EGb761 exerts also anti-tumorigenic properties by reducing cell viability and proliferation while concomitantly inducing apoptosis in hepatoma cell lines (Figs 2 and 3).[37] Interestingly, we observed a differential response to exposure of EGb761 in normal and malignant cells of the liver. While concentrations of 200μg/ml already suppressed cell viability and induced apoptosis in hepatoma cell lines, untransformed hepatocytes were only affected by high doses of Ginkgo biloba (>500μg/ml) suggesting a different sensitivity of malignant and non-malignant cells to EGb761. Additionally, we observed that EGb761 significantly impaired the ability of hepatoma cells to form spheres and colonies (Fig 4). Notably, tumorigenic abilities of forming colonies and spheres were significantly inhibited only in hepatoma cells while comparable doses did not affect IH indicating that EGb761 preferentially affects transformed cells and could be safely used as a complementary treatment strategy and/or to reduce side-effects. These observations are in line with recent studies that confirmed preventive properties and demonstrated pro-apoptotic as well as anti-proliferative effects of EGb761 in a variety of cancers including HCC.[37–39] EGb761 decreased cell migration and survival as well as tumor progression through anti-proliferative, anti-angiogenic, anti-oxidant, and apoptosis-inducing activities *in vitro* and *in vivo* [38, 40, 41]. Further, EGb761 also exerted synergistic effects in combination with systemic chemotherapies in advanced tumors of the gastro-intestinal tract.[42–44] Tolerability and safety could be also confirmed for the combination therapy of Ginkgo biloba and sorafenib in patients with advanced HCCs.[45]

Consistent with our results, EGb761 at a concentration of 400μg/ml induced apoptosis in a panel of melanoma cells. Mechanistically, EGb761 triggered activation of the mitochondrial apoptotic pathway by an imbalance of pro- and anti-apoptotic proteins of the Bcl-2 family. Notably Mcl-1 played a major role in EGb761-induced apoptosis in melanoma cells. Interestingly and consistent with our here presented results, the study also revealed that EGb761 had no meaningful effects on untransformed melanocytes. However, the underlying molecular mechanism remained unclear.[21] Our global gene expression analyses demonstrated that key oncogenic pathways in hepatocarcinogenesis involved in cell proliferation and survival, e.g. PI3K/AKT/mTOR, MAPK/ERK, IGF-1, as well as Nrf2-mediated oxidative and xenobiotic stress response are significantly regulated by the EGb761 treatment in hepatoma cells (Fig 5 and Table 1). A recent study indicates that Gingko biloba might inhibit AKT/mTOR signaling and reduce activation of p70S6K whereby preventing renal fibrosis.[46] The PI3K/AKT/mTOR pathway is believed to be a major pathway regulating mRNA translation by activating of p70SK6 and several initiation factors.[47] During malignant transformation and cancer progression, cancer cells require increased rates of protein synthesis for growth and metabolic reprogramming.[48] It is generally accepted that initiation of mRNA translation is the rate-limiting step for protein synthesis and frequently deregulated in human cancers thereby contributing to uncontrolled growth and survival.[49] Our data suggests that a deregulation of EGb761-regulated oncogenic pathways in hepatoma cells, e.g. mTOR-signaling, as a potential reason for a different response of malignant and non-malignant cells to EGb761 (Table 1, Figs 5 and 6) ultimately resulting in a disruption of cancer cell survival by impaired protein biosynthesis.

**Table 1:** Comparison analysis of canonical pathways identified by Ingenuity Pathway Analysis.

Canonical Pathways	Cell line	p-value	No. of genes	Genes
Xenobiotic Metabolism Signaling	Huh7	2,95E-02	7	CYP1B1, MAP3K3, **MGST1**, PIK3CB, PPP2R5B, PPP2R5D, SCAND1
	WRL68	6,88E-04	25	ABCC2, ALDH16A1, **ALDH1A3**, ALDH3A2, CHST15, **EIF2AK3, GSTM1, GSTM3**, GSTP1, *HDAC4*, **HMOX1, MAP3K8, MGST1**, NDST1, *NFE2L2*, NQO1, NQO2, *NRAS*, ***NRIP1***, PIK3R4, PPP2R4, *RRAS2*, RXRA, ***UGT1A3***, UST
	Pitts1	n.s.	4	MAP2K1, *NDST2*, ***NRIP1***, **UGT1A3**
	THLE5B	7,27E-03	17	**ALDH1A3**, ALDH7A1, CAT, *CITED2*, **EIF2AK3, *GSTM1*, *GSTM3*, *HMOX1***, *IL6*, MAOA, MAP3K6, ***MAP3K8***, MED1, MRAS, *PIK3R3*, PRKCA, PRKCH
Nrf2-mediated Oxidative Stress Response	Huh7	4,69E-02	5	***DNAJB4***, GCLM, **MGST1**, PIK3CB, **UBB**
	WRL68	1,77E-07	26	ABCC2, ACTA2, *ATF4*, *DNAJA3*, **DNAJB4**, DNAJC13, EIF2AK3, EPHX1, FTH1, **GSTM1, GSTM3**, GSTP1, **HMOX1**, MAFG, **MGST1**, *NFE2L2*, NQO1, NQO2, *NRAS*, PIK3R4, PRDX1, *RRAS2*, SLC3A2, SQSTM1, TXNRD1, VCP
	Pitts1	n.s.	4	DNAJB11, *JUNB*, MAP2K1, ***UBB***
	THLE5B	2,12E-03	14	CAT, *DNAJA1*, DNAJA2, *EIF2AK3*, ***GSTM1*, *GSTM3*, *HMOX1***, *JUN*, MRAS, PIK3R3, PRKCA, PRKCH, **SLC35A2**, **VCP**
PI3K/AKT Signaling	Huh7	4,53E-02	4	*GRB2*, PIK3CB, PPP2R5B, PPP2R5D
	WRL68	2,44E-02	11	BCL2L1, *EIF4EBP1*, *FOXO1*, IKBKE, **INPPL1, MAP3K8**, *NRAS*, PPP2R4, PTGS2, *RRAS2*, SFN
	Pitts1	n.s.	2	MAP2K1, *NFKBIA*
	THLE5B	1,80E-02	9	CHUK, ***INPPL1***, ITGA5, JAK2, ***MAP3K8***, MRAS, *PIK3R3*, SHC1, *YWHAH*
Regulation of eIF4 and p70S6K	Huh7	2,17E-02	5	*GRB2*, PIK3CB, PPP2R5B, PPP2R5D, ***RPS12***
	WRL68	2,68E-03	15	*AGO3*, *EIF3H*, **EIF3L**, *EIF3M*, *EIF4A1*, *EIF4EBP1*, EIF4G3, *NRAS*, PIK3R4, PPP2R4, *RPS3*, ***RPS****12*, RPS28, *RPS15A*, *RRAS2*
	Pitts1	6,28E-03	5	EIF3D, *EIF3F*, MAP2K1, RPS29, *RPS4X*
	THLE5B	n.s.	5	***EIF3L***, ITGA5, MRAS, *PIK3R3*, SHC1
Estrogen Receptor Signaling	Huh7	n.s.	3	*GRB2*, ***MED10***, POLR2J2/POLR2J3
	WRL68	3,15E-02	11	*GTF2H3*, MED24, *MED13L*, *MNAT1*, *NRAS*, ***NRIP1***, *POLR2C*, *POLR2G*, *POLR2H*, *RRAS2*, *TAF9*
	Pitts1	8,75E-06	8	DDX5, *H3F3A/H3F3B*, *HIST2H3C*, MAP2K1, *MED12*, *NRIP1*, *PCK2*, POLR2I
	THLE5B	2,27E-02	9	*CCNH*, MED1, **MED10**, MED17, MRAS, SHC1, *TAF15*, TAF1L, TAF9B
Glucocorticoid Receptor Signaling	Huh7	n.s.	6	***BGLAP***, *CDKN1C*, *GRB2*, NFTC3, PIK3CB, POLR2J2/POLR2J3
	WRL68	3,71E-03	23	BCL2L1, ***BGLAP***, *BRD7*, CCL2, *GTF2H3*, *HLTF*, HSPA1A/HSPA1B, IKBKE, *MNAT1*, *NRAS*, ***NRIP1***, PIK3R4, *POLR2C*, *POLR2G*, *POLR2H*, PTGS2, *RRAS2*, *SMAD4*, STAT1, STAT3, STAT5A, *TAF9*, TRAF2
	Pitts1	2,29E-02	6	MAP2K1, *NFKBIA*, ***NRIP1***, *PCK2*, POLR2I, *SMARCD2*
	THLE5B	3,36E-06	25	*ADRB2*, *AGT*, *CCNH*, CHUK, *CXCL8*, *DUSP1*, *ELK1*, FKBP4, *IL6*, JAK2, *JUN*, MED1, MRAS, NR3C2, *PIK3R3*, *PPP3CB*, SERPINE1, *SGK1*, SHC1, *SLPI*, *TAB1*, *TAF15*, TAF1L, TAF9B, *YWHAH*

Shown are canonical pathways significantly regulated by EGb761 (p<0.05) in at least three of the four cell lines detected by a comparison analysis of Ingenuity Pathway Analysis. Up-regulated genes are presented in cursive and down-regulated genes in non-cursive letters. Genes in bold letters overlap in at least two cell lines.

Taken together, results of this study indicate that EGb761 has pleiotropic functional and molecular effects on hepatoma cells and IH. In particular, we observed that EGb761 differentially affects tumorigenic properties of malignant and non-malignant cells in the liver by interacting with multiple molecular pathways. Key oncogenic pathways resembling mTOR-signaling are differentially affected in malignant and non-malignant cells possibly due to a deregulation of the signaling pathways during malignant transformation. While the causal regulatory effects of EGb761 on these different signaling pathways need to be established in future investigations, these results indicate that EGb761 can be safely used for both preventive as well as therapeutic approaches in the liver and warrant future clinical investigations.

## Supporting information

S1 Table Top molecular and cellular functions identified by Ingenuity Pathway Analysis in hepatoma cell lines and human hepatocytes. (PDF)

S1 Fig Transcriptional changes by EGb761 in hepatoma cell lines and human hepatocytes. Transcriptomic changes to controls (untreated cells) after 24h, 48h and 72h EGb761 treatment of Keap1, Nfe2l2, Mapk1 and mTOR are demonstrated. The data are means ±SD of three independent experiments.
(TIF)

S2 Fig Full-length membranes of western blotting. Membranes of full-length western blotting (WB) of (p-)AKT, (p-)mTOR, (p-)ERK1/2, Nrf2, Keap1 and corresponding beta-Actin of untreated (-) and 24h, 48h and 72h EGb761 treated (+) THLE5B and Pitts1 cells.
(PDF)
