# Supplementary material for: Ginkgo biloba induces different gene expression signatures and oncogenic pathways in malignant and non-malignant cells of the liver
Source: PLoS One. 2018 Dec 21;13(12):e0209067. doi: 10.1371/journal.pone.0209067 (PMC6303069; doi:10.1371/journal.pone.0209067)
Supplement: S1 Table — (PDF) [file pone.0209067.s001.pdf]

## Huh7

| Top Molecular and Cellular Functions | | |
|---|---|---|
| **Name** | **p-Value Range** | **# Molecules** |
| **Cellular Function and Maintenance** | 3,00E-02 - 1,17E-04 | 31 |
| **Cellular Movement** | 2,16E-02 - 1,17E-04 | 10 |
| **Amino Acid Metabolism** | 2,99E-02 - 1,89E-04 | 12 |
| **Small Molecule Biochemistry** | 2,99E-02 - 1,89E-04 | 31 |
| **Vitamin and Mineral Metabolism** | 1,09E-02 - 2,29E-04 | 5 |

## WRL68

| Top Molecular and Cellular Functions | | |
|---|---|---|
| **Name** | **p-Value Range** | **# Molecules** |
| **Cell Death and Survival** | 1,35E-02 - 1,06E-08 | 303 |
| **Cellular Growth and Proliferation** | 1,18E-02 - 8,89E-07 | 309 |
| **RNA Post-Transcriptional Modification** | 6,49E-03 - 1,6E-06 | 38 |
| **Cellular Compromise** | 1,16E-02 - 4,88E-06 | 47 |
| **Protein Synthesis** | 1,16E-02 - 9,28E-06 | 91 |

## Pitts1

| Top Molecular and Cellular Functions | | |
|---|---|---|
| **Name** | **p-Value Range** | **# Molecules** |
| **Gene Expression** | 1,58E-02 - 5,21E-09 | 61 |
| **Cellular Growth and Proliferation** | 2,00E-02 - 2,43E-06 | 72 |
| **Cell Cycle** | 1,58E-02 - 1,33E-04 | 18 |
| **Cell Death and Survival** | 1,97E-02 - 1,86E-04 | 58 |
| **Protein Synthesis** | 1,05E-02 - 2,26E-04 | 22 |

## THLE5B

| Top Molecular and Cellular Functions | | |
|---|---|---|
| **Name** | **p-Value Range** | **# Molecules** |
| **Cellular Development** | 5,72E-03 - 3,17E-07 | 216 |
| **Cellular Growth and Proliferation** | 5,72E-03 - 6,97E-07 | 257 |
| **Cell Death and Survival** | 5,67E-03 - 1,37E-06 | 221 |
| **Cellular Function and Maintenance** | 5,72E-03 - 1,56E-06 | 193 |
| **Protein Synthesis** | 9,47E-04 - 7,09E-06 | 70 |
