# Supplementary figures and images for: Ginkgo biloba induces different gene expression signatures and oncogenic pathways in malignant and non-malignant cells of the liver
Source: PLoS One. 2018 Dec 21;13(12):e0209067. doi: 10.1371/journal.pone.0209067 (PMC6303069; doi:10.1371/journal.pone.0209067)

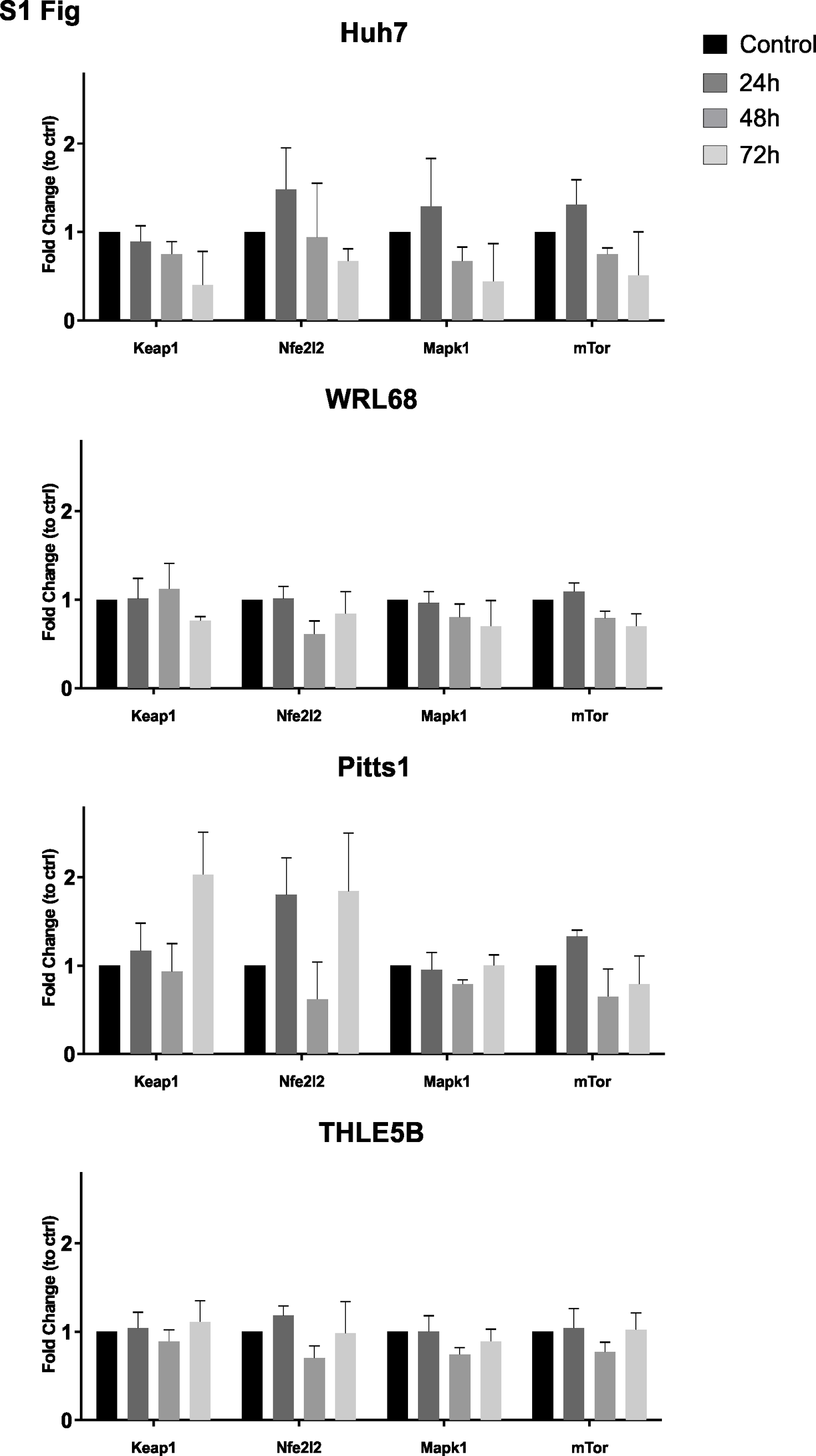

Supplement: S1 Fig — Transcriptomic changes to controls (untreated cells) after 24h, 48h and 72h EGb761 treatment of Keap1, Nfe2l2, Mapk1 and mTOR are demonstrated. The data are means ±SD of three independent experiments. (TIF) [file pone.0209067.s002.tif]
