# Supplementary material for: Ginkgo biloba induces different gene expression signatures and oncogenic pathways in malignant and non-malignant cells of the liver
Source: PLoS One. 2018 Dec 21;13(12):e0209067. doi: 10.1371/journal.pone.0209067 (PMC6303069; doi:10.1371/journal.pone.0209067)
Supplement: S2 Fig — Membranes of full-length western blotting (WB) of (p-)AKT, (p-)mTOR, (p-)ERK1/2, Nrf2, Keap1 and corresponding beta-Actin of untreated (-) and 24h, 48h and 72h EGb761 treated (+) THLE5B and Pitts1 cells. (PDF) [file pone.0209067.s003.pdf]

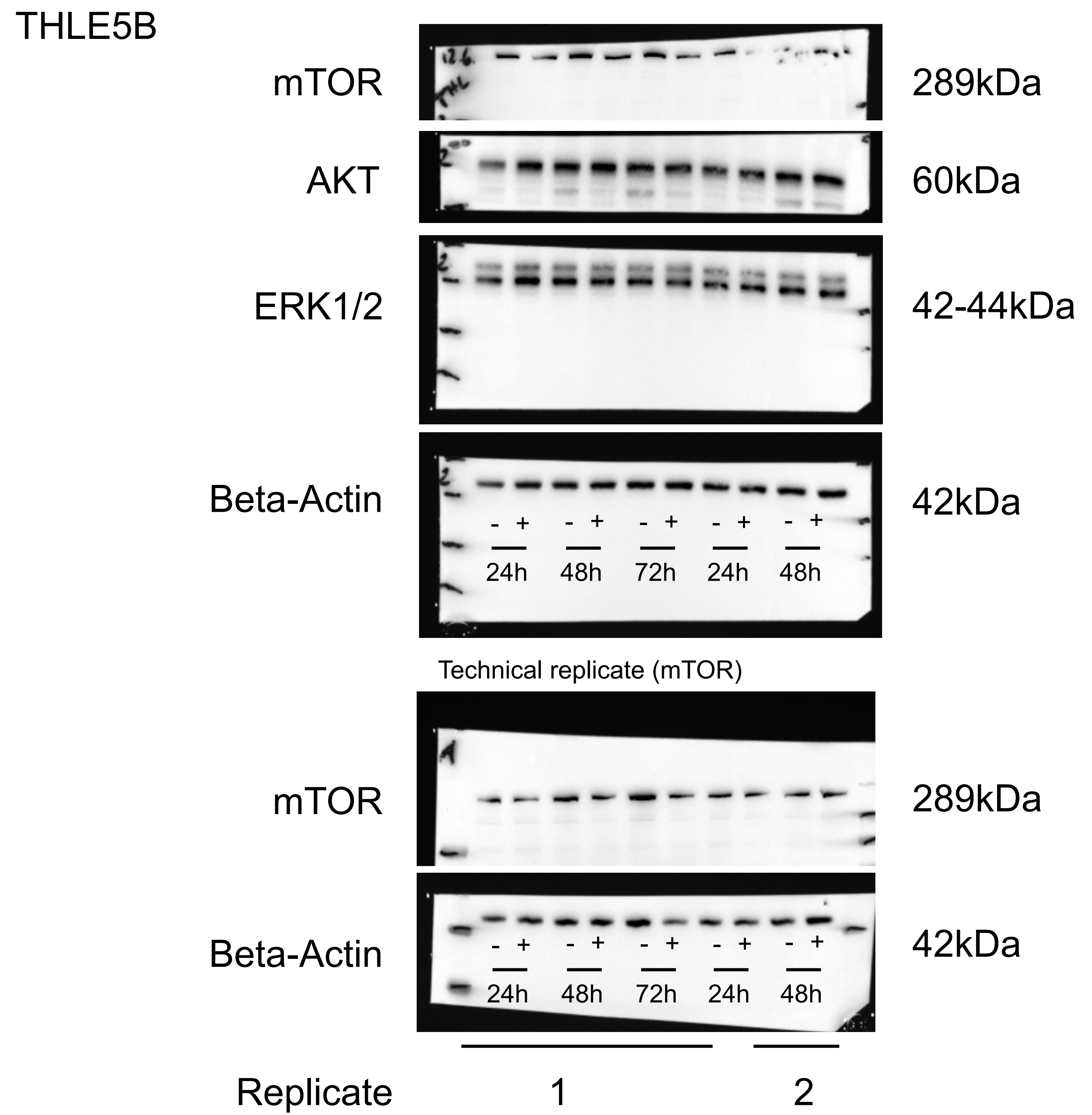
THLE5B
mTOR
289kDa
AKT
60kDa
ERK1/2
42-44kDa
Beta-Actin
42kDa
- + - + - + - + - +
24h 48h 72h 24h 48h
Technical replicate (mTOR)
mTOR
289kDa
Beta-Actin
42kDa
- + - + - + - + - +
24h 48h 72h 24h 48h
Replicate
1
2

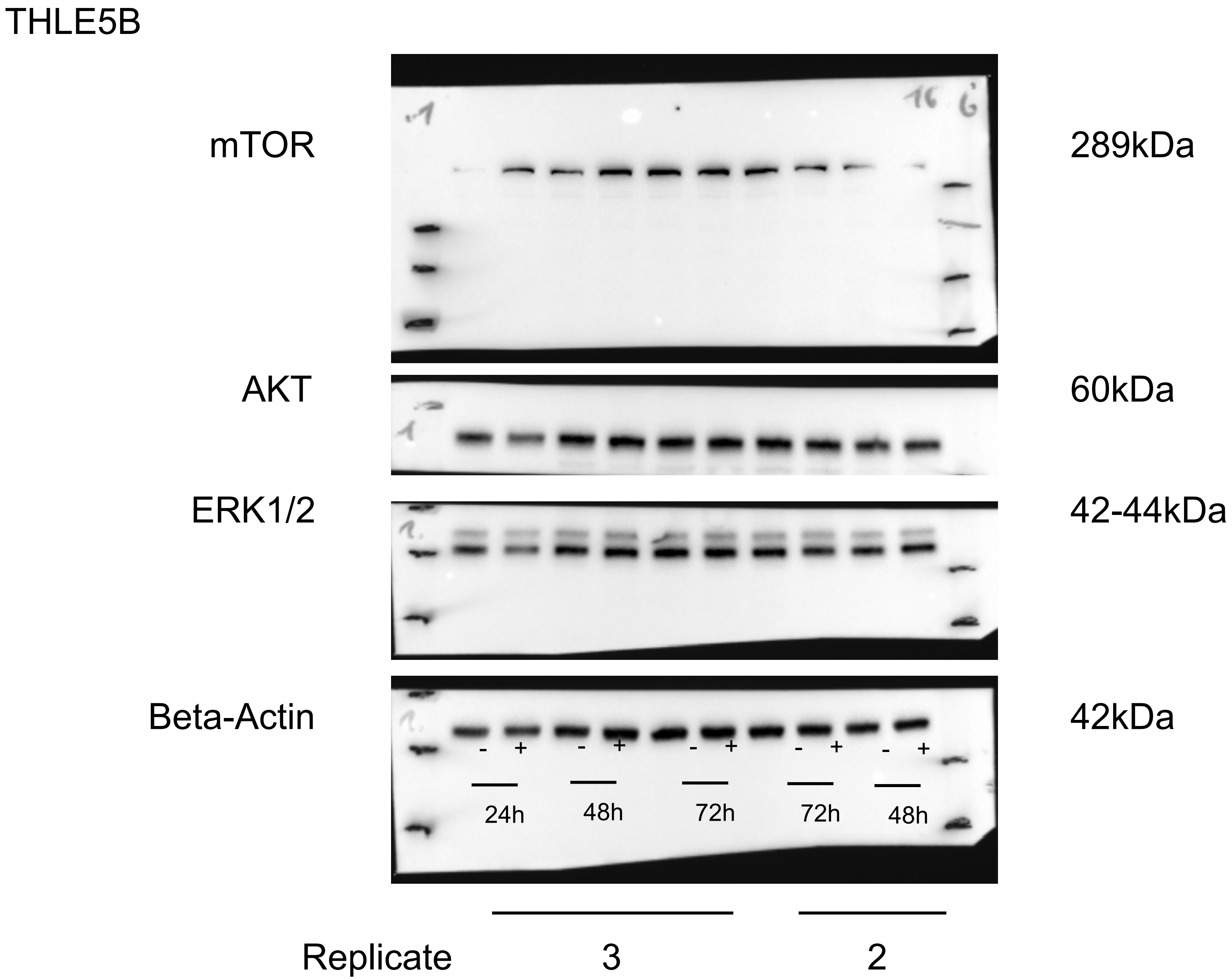

THLE5B
mTOR
289kDa
AKT
60kDa
ERK1/2
42-44kDa
Beta-Actin
42kDa
- + - + - + - + - +
24h
48h
72h
72h
48h
Replicate
3
2

THLE5B

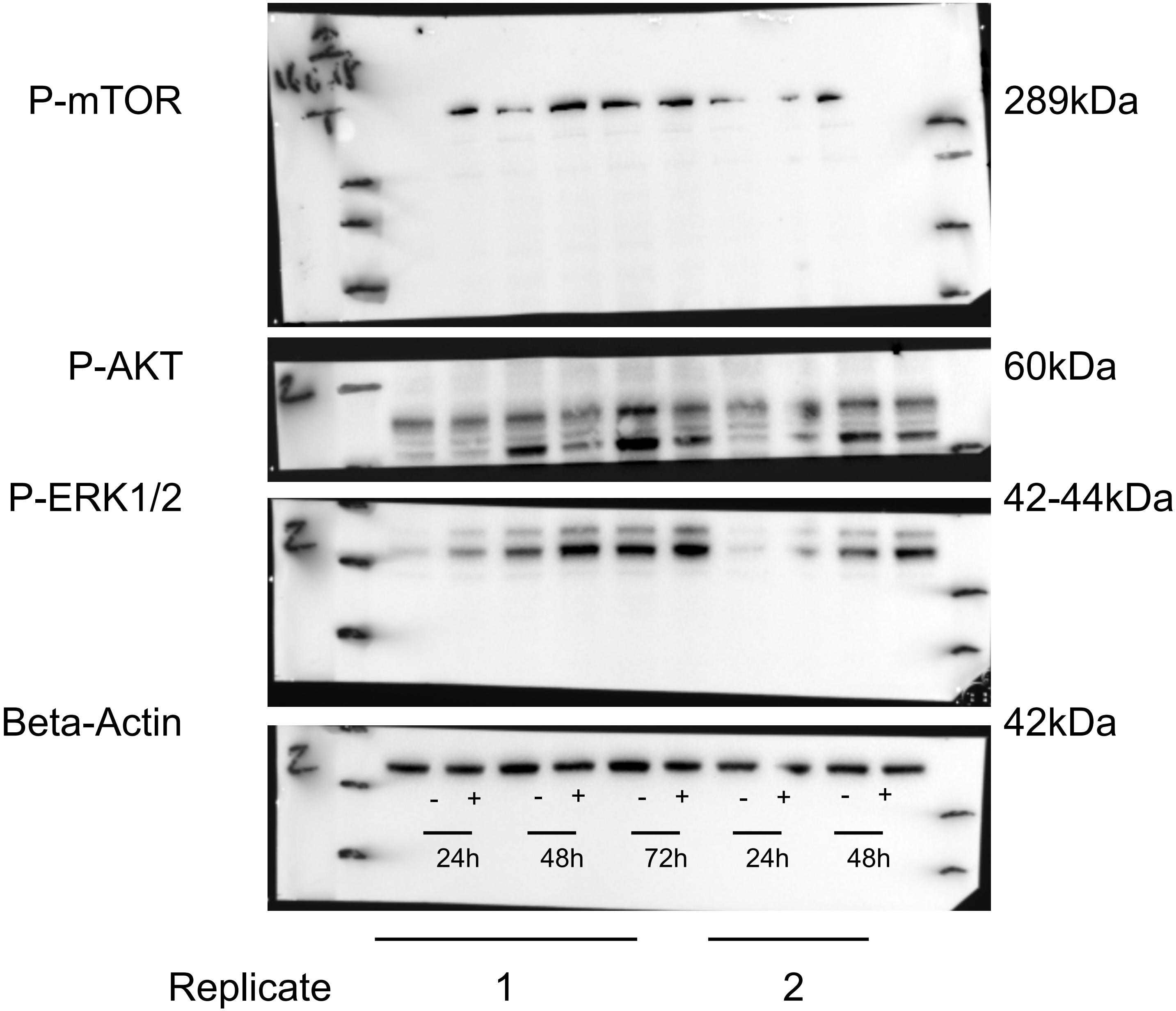

P-mTOR
289kDa
P-AKT
60kDa
P-ERK1/2
42-44kDa
Beta-Actin
42kDa
- + - + - + - + - +
24h 48h 72h 24h 48h
Replicate 1 2

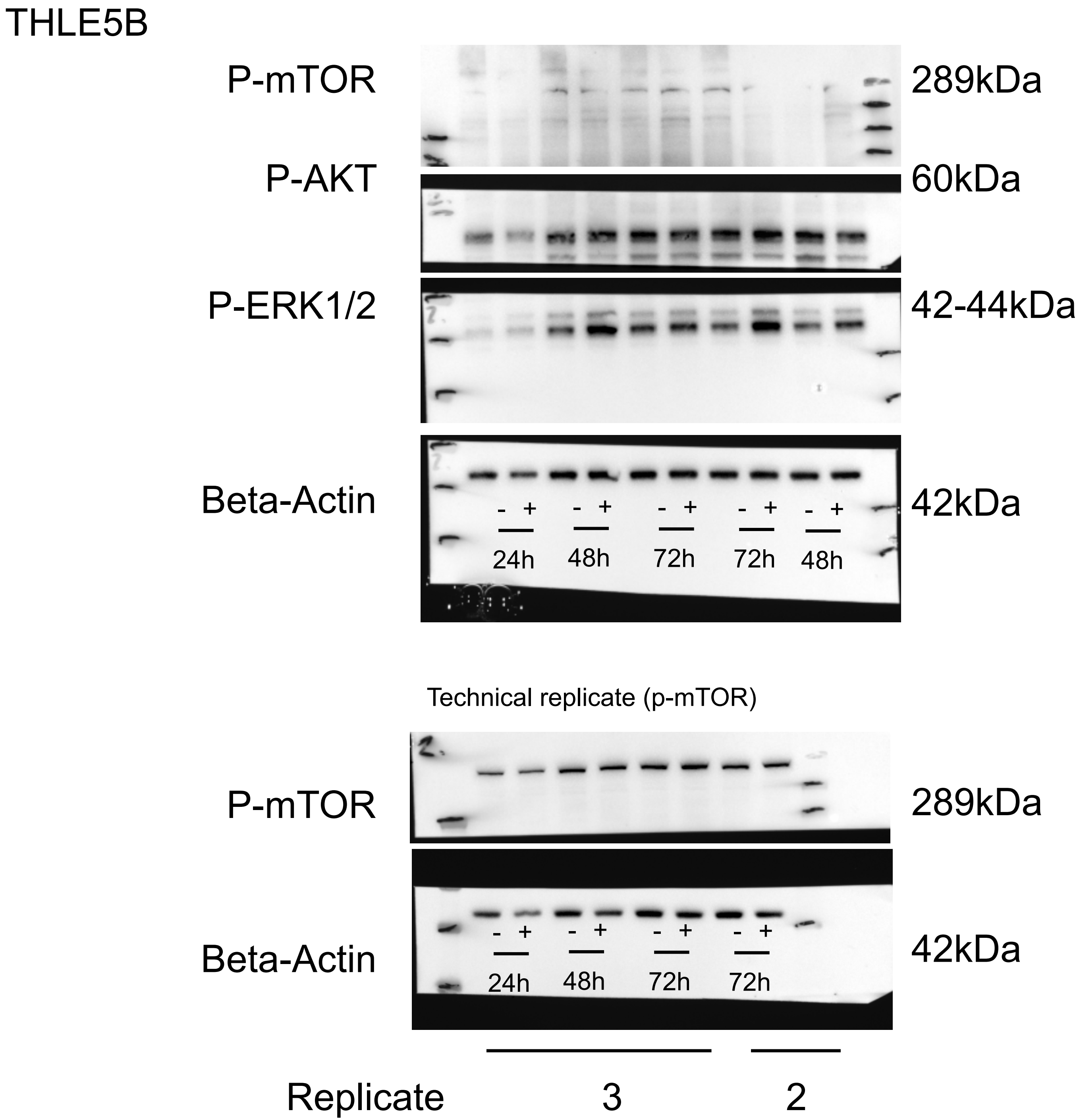
THLE5B
P-mTOR
289kDa
P-AKT
60kDa
P-ERK1/2
42-44kDa
Beta-Actin
42kDa
- + - + - + - + - +
24h 48h 72h 72h 48h
Technical replicate (p-mTOR)
P-mTOR
289kDa
Beta-Actin
42kDa
- + - + - + - +
24h 48h 72h 72h
Replicate 3 2

THLE5B

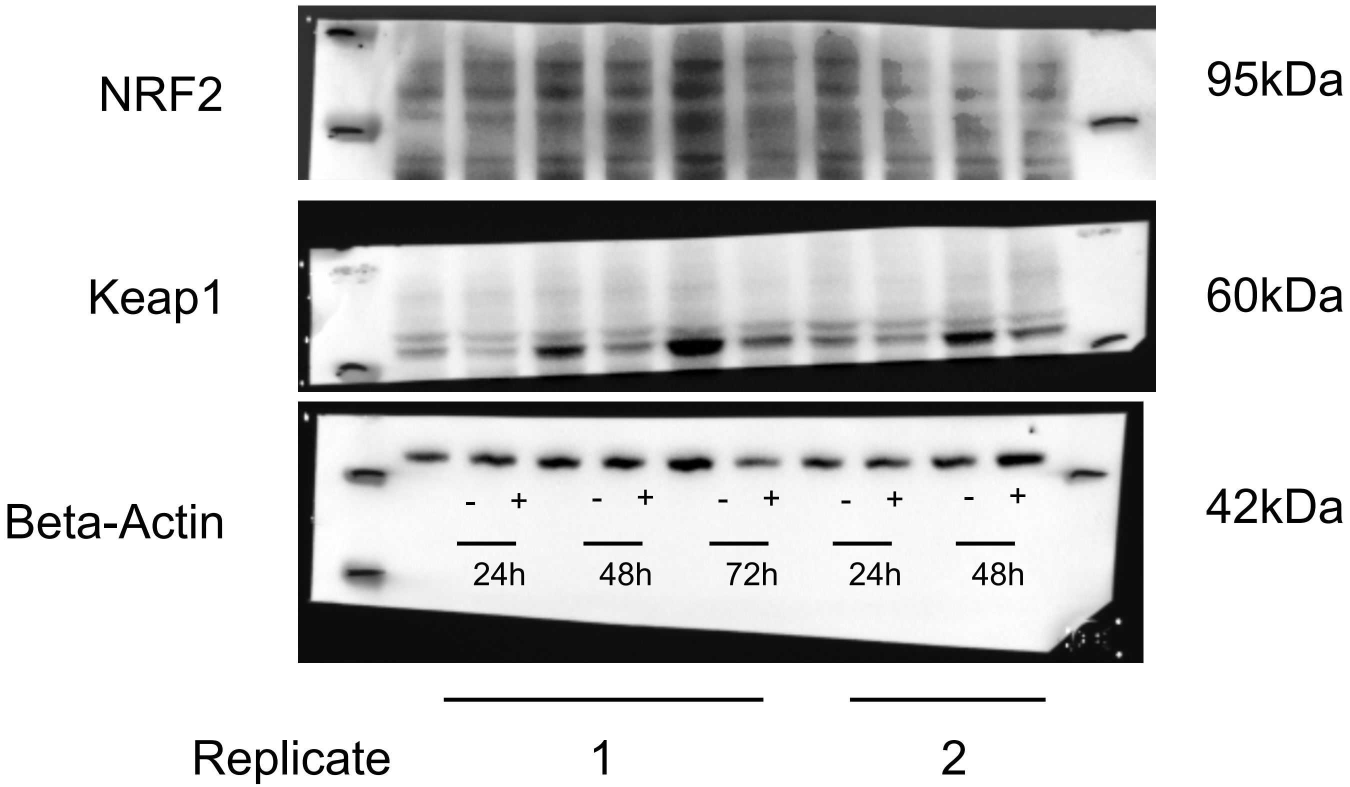
NRF2
95kDa
Keap1
60kDa
Beta-Actin
42kDa
- + - + - + - + - +
24h 48h 72h 24h 48h
Replicate 1 2

THLE5B

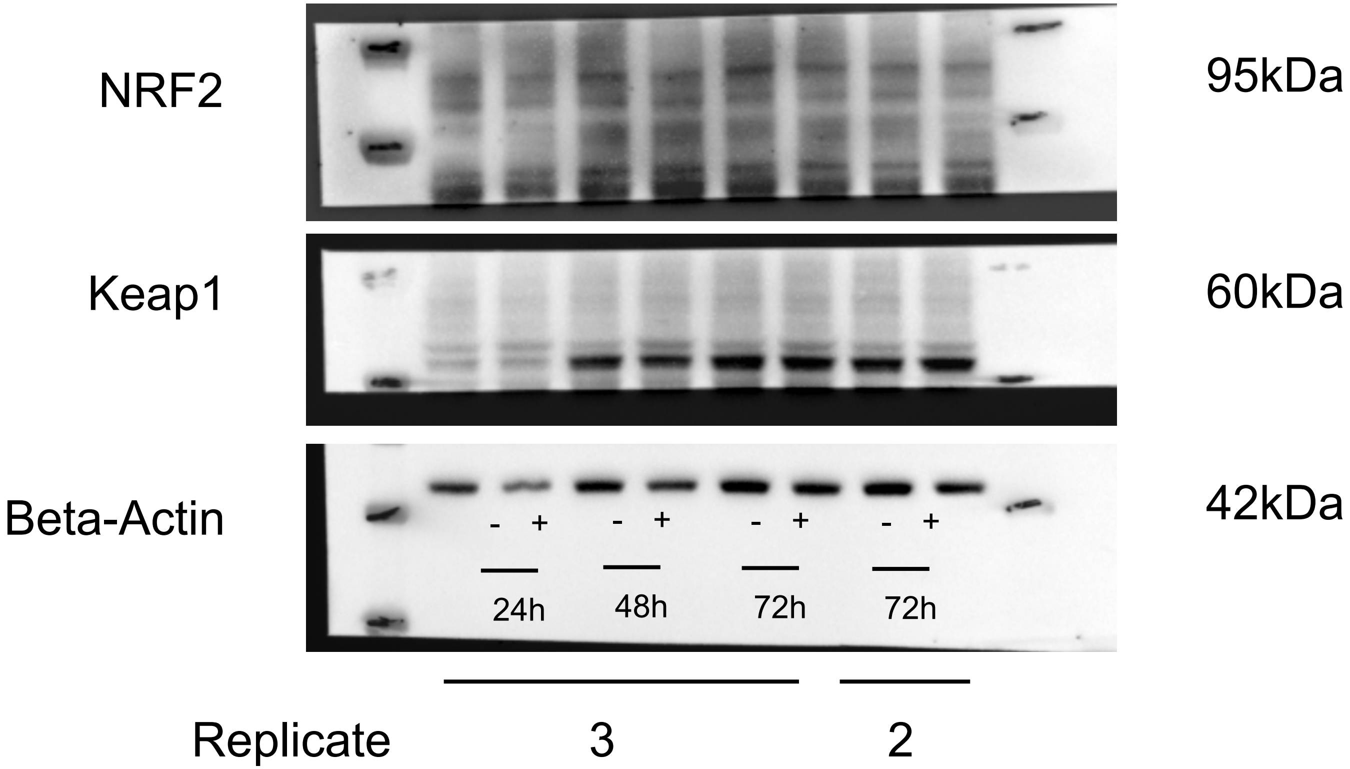
NRF2
Keap1
Beta-Actin
95kDa
60kDa
42kDa
- +
- +
- +
- +
24h
48h
72h
72h
Replicate
3
2

Pitts1

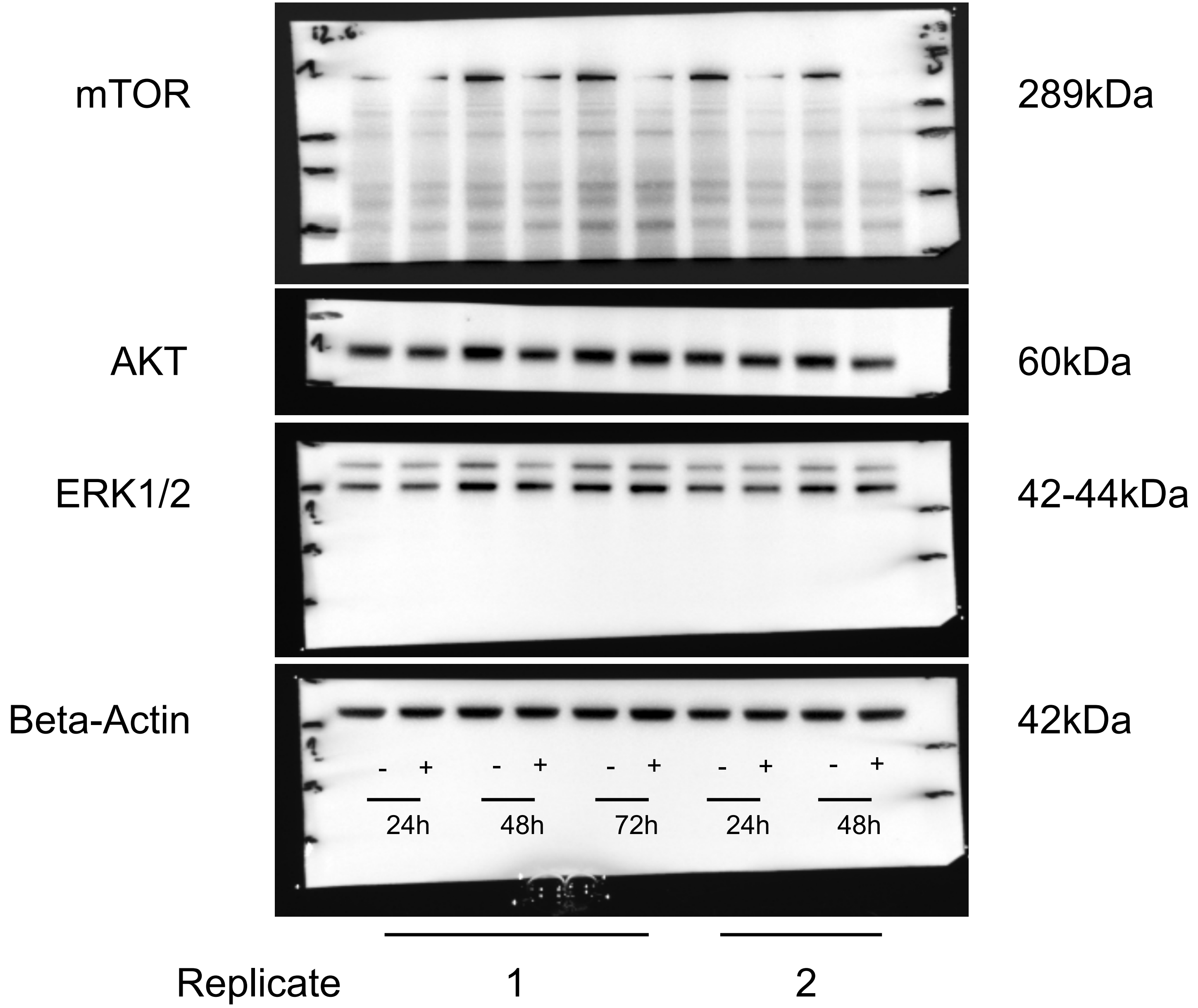
mTOR
289kDa
AKT
60kDa
ERK1/2
42-44kDa
Beta-Actin
42kDa
- + - + - + - + - +
24h 48h 72h 24h 48h
Replicate
1
2

Pitts1

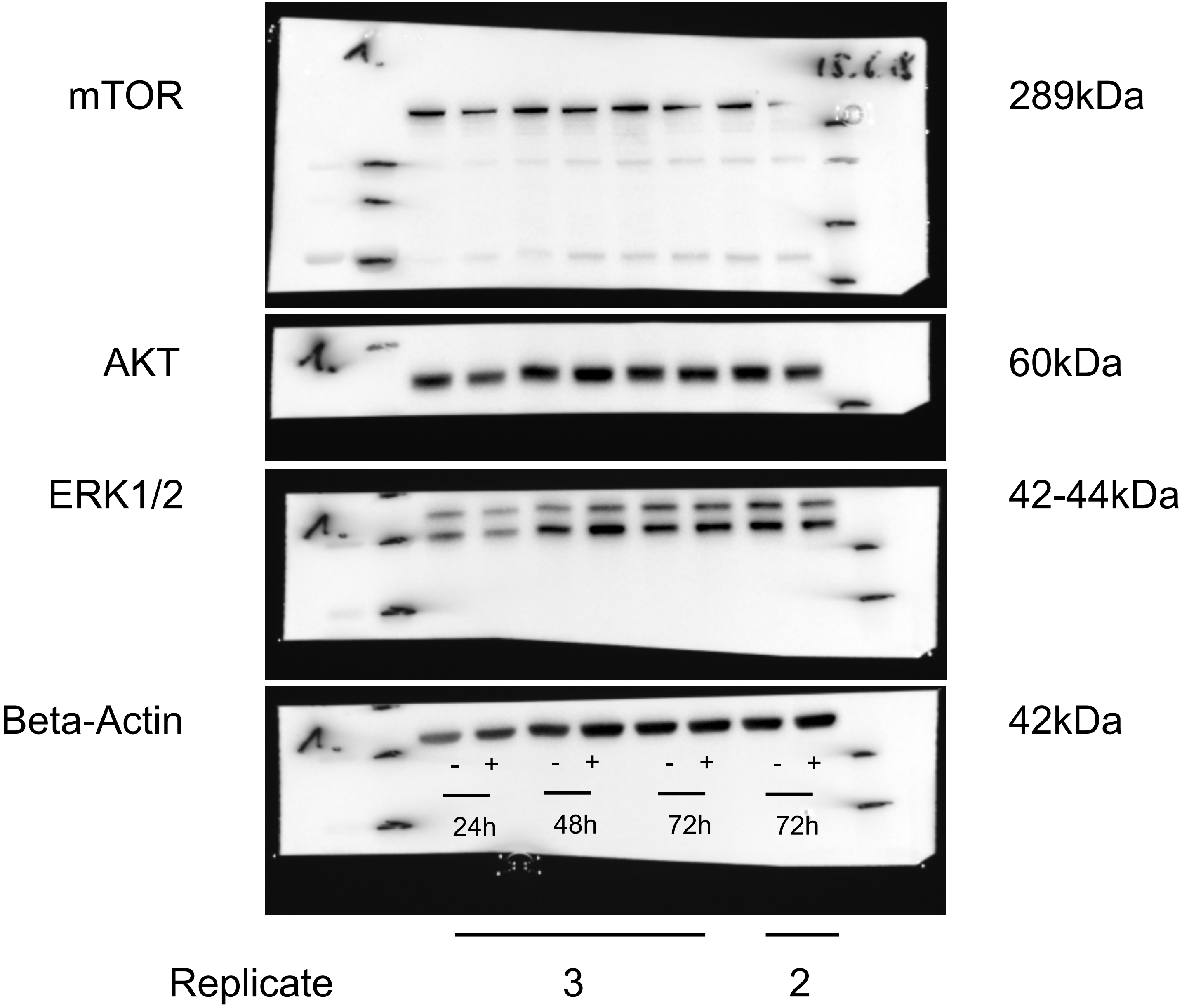
mTOR
289kDa
AKT
60kDa
ERK1/2
42-44kDa
Beta-Actin
42kDa
- + - + - + - +
24h
48h
72h
72h
Replicate
3
2

Pitts1

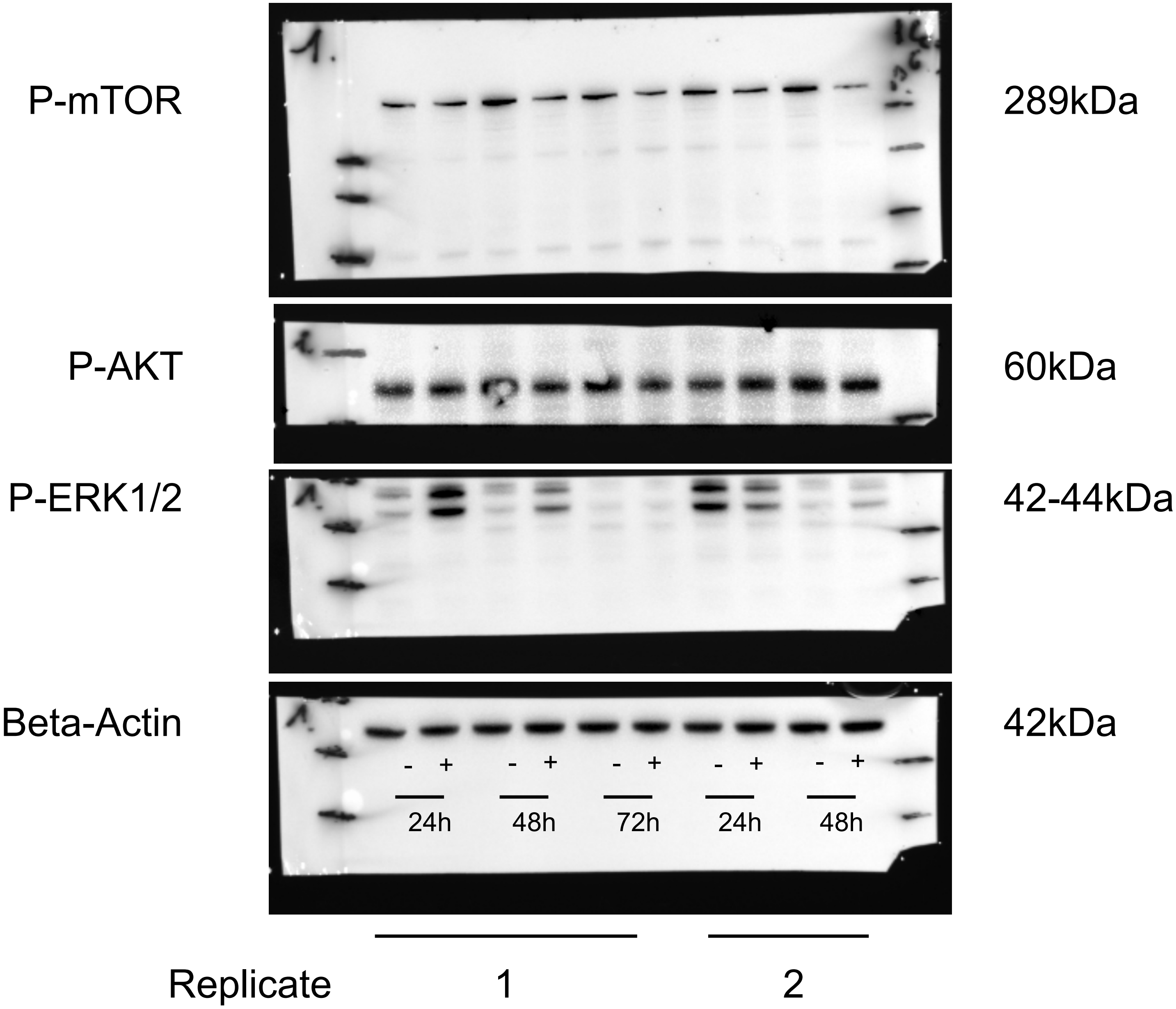
P-mTOR
289kDa
P-AKT
60kDa
P-ERK1/2
42-44kDa
Beta-Actin
42kDa
- + - + - + - + - +
24h 48h 72h 24h 48h
Replicate 1 2

Pitts1

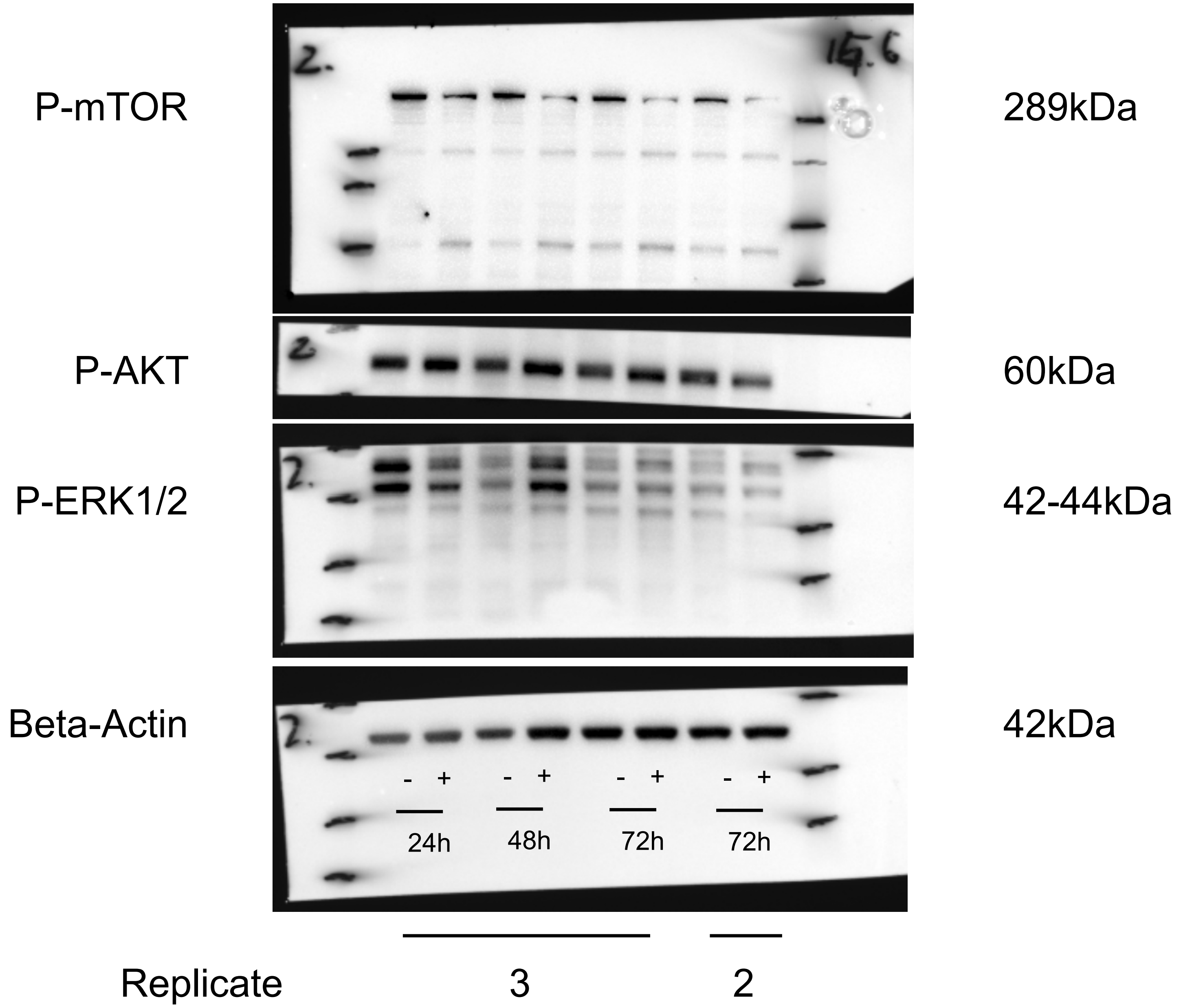
P-mTOR
289kDa
P-AKT
60kDa
P-ERK1/2
42-44kDa
Beta-Actin
42kDa
- + - + - + - +
24h 48h 72h 72h
Replicate 3 2

Pitts1

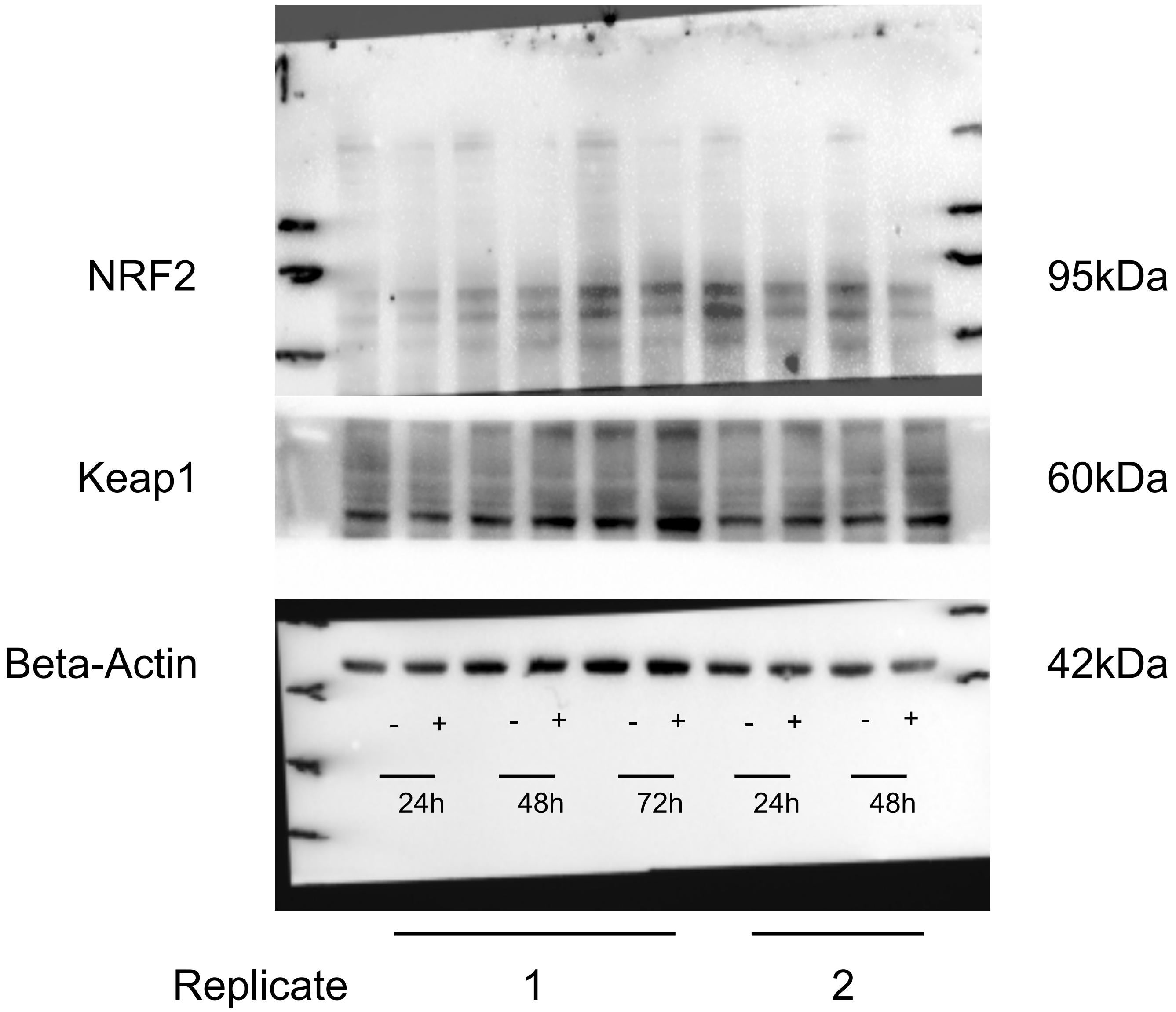
NRF2
95kDa
Keap1
60kDa
Beta-Actin
42kDa
- + - + - + - + - +
24h 48h 72h 24h 48h
Replicate
1
2

Pitts1

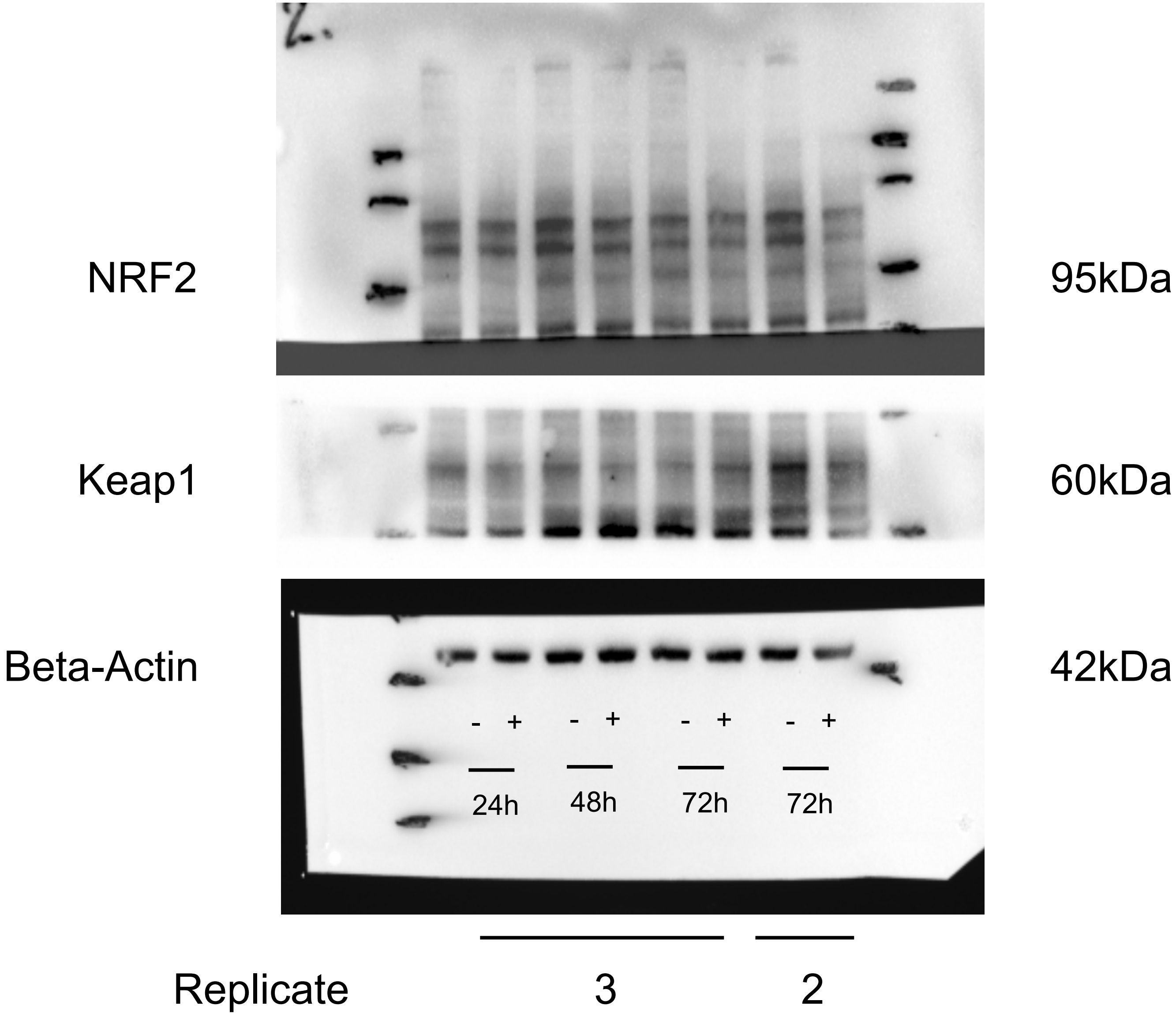

NRF2
95kDa
Keap1
60kDa
Beta-Actin
42kDa
- + - + - + - +
24h 48h 72h 72h
Replicate 3 2
